# A Scoping Review of Influences on HPV Vaccine Uptake in the Rural US †

**DOI:** 10.3390/vaccines14020156

**Published:** 2026-02-05

**Authors:** Sherri Sheinfeld Gorin, Rebecca Hyman, Courtney Olson, Elizabeth Amber Fournier, Kaitlyn Yang, Diana Hanko, HPV Review Working Group

**Affiliations:** 1 Department of Family Medicine, School of Medicine, University of Michigan, Ann Arbor, MI 48104, USA; 2 School of Public Health, University of Michigan, Ann Arbor, MI 48104, USA; 3 Albert Einstein College of Medicine, Bronx, NY 10461, USA; rebecca.hyman@einsteinmed.edu; 4 University of Iowa, Iowa City, IA 52242, USA; olsonco@umich.edu; 5 University of Michigan College of Literature, Science, and the Arts, Ann Arbor, MI 48109, USA; efourn@umich.edu (E.A.F.); kaitlyny@umich.edu (K.Y.); 6 Michigan State University, Lansing, MI 48824, USA; dhanko@umich.edu

**Keywords:** HPV vaccine, rural, scoping review, uptake

## Abstract

The human papillomavirus (HPV) is the leading cause of cervical and oropharyngeal cancers. Vaccination can prevent over 90% of HPV-attributed cancers. Rural populations are less likely to initiate and complete HPV vaccinations than urban. The primary objective of this paper is to systematically examine the multilevel (child/youth, parent/caregiver, physician/team, healthcare organization, community, and policy) influences on HPV vaccine uptake in the rural US population. As a secondary aim, we seek to identify gaps in the research that could contribute to the development of more precise intervention approaches in this population. The study adds to the limited number of recent reviews on rural HPV vaccine uptake in the US. Method: We conducted a systematic search of published empirical studies over 13 years (2010–2023), resulting in 1657 publications. The following databases were searched: Medline (OVID), Embase, CINAHL, PsychInfo, Cochrane, Sociological Abstracts, and Scopus using pre-specified inclusion criteria. Two reviewers independently coded 101 full texts; discrepancies were resolved by a third reviewer. The primary outcome was HPV vaccine uptake. Results: Adolescents themselves were the most common foci of change. Barriers to rural HPV uptake included limited; vaccine awareness, access to vaccines for children vaccination sites, and primary care recommendations. Conclusions: Tailored interventions to rural parents/caregivers could increase uptake of the vaccine. Provider training increases HPV vaccine recommendations; programs should also be targeted to rural school nurses, pharmacists, and dental care providers. Linking primary care practices and public health dissemination strategies are key.

## 1. Introduction

The human papillomavirus (HPV) is the most common sexually transmitted infection. The majority of cervical cancers are caused by persistent infections with oncogenic or high-risk types of human papillomavirus (HPV) [1]. Further, oncogenic forms of the virus cause a subset of oropharyngeal (oral cavity and pharynx) cancer, which is increasing in incidence [2]; men are more than twice as likely as women to be diagnosed [3,4,5,6,7]. The HPV vaccine is effective at preventing more than 90% of HPV infection-associated cancers. The HPV vaccine is effective at preventing more than 90% of HPV infection-associated cancers [1]. The Advisory Committee on Immunization Practices (ACIP) has recommended routine administration of the HPV vaccine for 11–12 year olds, as early as age 9, and catch-up vaccines through age 26 [8]. HPV vaccination rates remain below the Healthy People 2030 target of 80% [9]. Vaccination rates in rural communities are consistently lower than in urban communities in the rest of the country [10,11,12,13]. Among adolescents between the ages of 13 and 17 in 2024, 81.7% received more than one dose of the HPV vaccine in metropolitan areas, compared to 71.2% in mostly rural non-metropolitan areas. Additionally, 65.6% of 13–17 year olds in mostly urban areas were up-to-date with their HPV vaccine doses, compared to 54.8% in mostly rural areas. These variations in vaccination have led to higher mortality from HPV-associated cancers among rural residents [12,13].

Disparities in HPV vaccination rates among rural residents relative to urban and suburban residents are due to a number of factors. Allocations of healthcare and other resources across rural populations are impacted by the definition of rurality that is applied, and these definitions vary considerably [14,15]. Individual studies have found that travel time and low density of healthcare resources may pose challenges for accessing primary care to administer the vaccine in rural areas [16,17]. Some rural public health programs do not have resources to promote HPV vaccination or to devote to immunization in general [18]. These resources—as well as HPV vaccination messages—may not be well coordinated [17,19]. Many children in rural areas are seen by family practice providers whose panel includes very few adolescents and, thus, may not be comfortable making strong recommendations that are key to reducing HPV vaccine hesitancy [20,21,22,23,24]. Lack of awareness, limited knowledge, fear, and community stigma may increase hesitancy among adolescents and their caregivers [25,26,27,28]. Structural barriers like limited access to care and lack of insurance coverage influence HPV vaccine uptake, particularly in rural areas [10,29,30]. These influences may vary across and within rural communities [31,32], for example, due to the diversity of residents, including migrant agricultural workers, and Native Americans [33,34,35].

Importantly, the COVID-19 pandemic dramatically changed the uptake of all vaccines, including those against HPV. Studies reported increased levels of HPV vaccination hesitancy, vaccine refusals, and overall lower uptake within certain communities in the United States, including in rural areas [36,37]. The pandemic created major disturbances for routine HPV vaccine administration that were not captured in many early studies, nor in early reviews [38].

The majority of the reviews published on HPV vaccine uptake over the past 13 years have been narrow in focus [10,29,30,39,40,41,42,43,44]. In particular, none of these reviews have described the influences on HPV vaccine uptake in rural subpopulations in the US, although they have included rural residents as study participants [30,40,41,42,43,44]. The earliest of these reviews by Peterson et al. showed that increased HPV knowledge, peer-influence factors, and receiving other vaccines have been associated with HPV vaccine initiation and completion in rural communities [10]. This scoping review limited its search criteria to barriers and facilitators of vaccination at the individual level. The scoping review provided limited data on organizational, community- level and societal factors for their effects on HPV vaccination.

Other earlier reviews of rural HPV vaccine uptake were narrow in scope, either focused on measures [29] or interventions alone [39]. A recent systematic review and meta analysis explored the contribution of healthcare worker (HCW) practices to HPV vaccine recommendations worldwide. The systematic review and meta analysis results found mixed levels of HCW HPV vaccine confidence that varied by geographic location and gender of the vaccine recipient, as well as by type of physician. Worldwide, HPV vaccine recommendations were less common in rural areas than in urban communities, and within countries without national programs, as in the US. To increase recommendations, they highlighted the need for more contextually relevant approaches to addressing HCW vaccine hesitancy, as in the multilevel model of this scoping review [40].

Another scoping review examined digital health interventions for HPV vaccination in the US; the review focused on racial and ethnic minority groups, rather than rural populations, however [42]. With limited broadband access to about 38% of rural communities, digital interventions have limited reach in these communities [41].

Several current systematic reviews, scoping reviews, and one meta analysis have examined increasing HPV vaccination rates and promoting interventions [30] worldwide, generally including the US. One of these systematic reviews examining childhood immunizations, including HPV, highlighted middle-and lower-income economies, although uptake in rural USA settings was not specified [43]. Another systematic review of worldwide HPV vaccinations excluded males [44]. The Escoffery et al. systematic review highlighted the need to expand the implementation of HPV vaccine promotion interventions beyond education alone and at a single level of intervention [30].

Only one review has systematically examined multilevel influences on rural HPV vaccination in the US. Multilevel influences (targeting the child/youth, parent/caregiver, physician/team, healthcare organization, community, and policy) are defined as “…change in the individual patient, as well as at least two levels of contextual influence.” Multilevel factors can influence change among children and youth, parents and caregivers, healthcare providers and teams, clinics and health systems, communities, and policies. This recent systematic umbrella review (review of reviews [45]) identified modifiable individual-, provider-, and clinic-level factors associated with HPV vaccination outcomes among U.S. adolescents and developed a multilevel framework illustrating relations between factors to inform intervention development. Our review advances this comprehensive paper by adding the community and policy levels to the scoping review. Further, as an umbrella review, quality problems and biases that might exist in primary studies and in the umbrella review process itself could be compounded and difficult to clarify [46].

An updated review of the empirical literature on rural HPV vaccine uptake is warranted to better understand the influences on lower vaccination rates. To our knowledge, no review has yet focused on HPV vaccine uptake in the rural US using a multilevel perspective that includes the community and policy levels. In accord with the aim of scoping reviews in general, we aim to provide an overview or map of the available evidence, rather than synthesized findings for implementation into policy or practice [47]. The primary objective of this paper is therefore to systematically examine the multilevel influences on HPV vaccine uptake in the rural US population. As a secondary aim, we seek to identify gaps in the research that could contribute to the development of more precise intervention approaches in this population.

## 2. Methods

We conducted a scoping literature review of factors influencing HPV vaccine uptake in the rural US between 2010 and 2023.

### 2.1. Search Strategy

A skilled informaticist searched seven databases (Medline (OVID), Embase, CINAHL, PsychInfo, Cochrane, Sociological Abstracts, and Scopus) using the inclusion criteria detailed below. The primary search was conducted in Medline (OVID), and MeSH terms were combined with keywords in the title, abstract, and author-supplied keywords. The other database searches were translations of that primary search and used the database’s controlled vocabulary when available (e.g., for EMBASE, we used EMTREE). MeSH is not always available in the other databases. Several “gold standard” articles were identified in advance; the search located each of them.

Key search terms included: HPV, virus, vaccination, measures, and rural (see Appendix A for the full search strategy).

**Figure 1:**
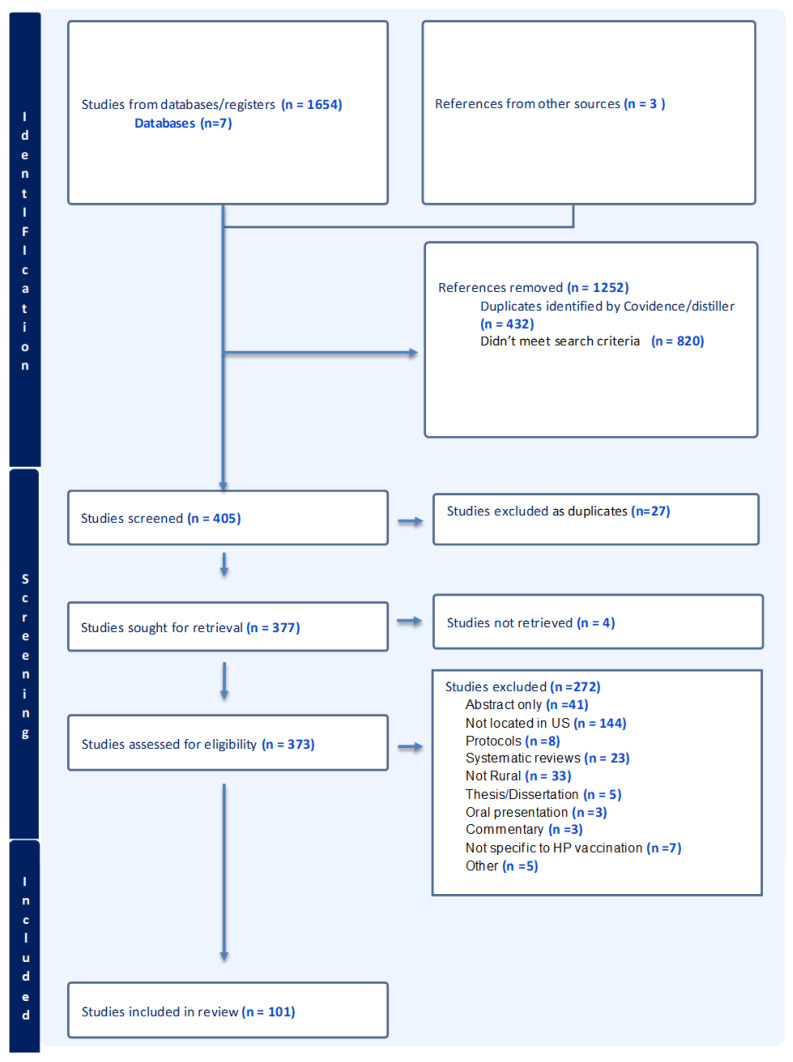
Study recruitment.

Two systematic searches were conducted by a skilled informaticist over time; the second search updated the first search. Identical search terms were used, and the same inclusion and exclusion criteria were applied to both searches. As depicted on the PRISMA table (see Figure 1), in total, 1657 articles were identified. These included the three papers that were identified from alternative sources, other than bibliographic databases and registries.

For the first search, articles were screened using the DistillerSR software version 2.35 [48] to eliminate duplicate articles and those that did not meet the specific search criteria. In the second search, the Covidence software [49] was used to deduplicate the articles, as well as to narrow the search. Both Distiller and Covidence software are similar in their key features [50]; the search findings were consistent; the choice was made by the informaticist, as the library changed its software recommendations over time [50]. After exclusions, 101 articles met the criteria to be included in this scoping review and were systematically coded (see Figure 1).

### 2.2. Source of Evidence Screening and Selection

We included original peer-reviewed, empirical articles that focused on HPV vaccine uptake, hesitancy behavior, attitudes/beliefs, and awareness/knowledge at multiple levels, including rural children and youth (age 9–26), families, healthcare providers, clinics, communities, or policies. HPV vaccine uptake was the primary outcome. Uptake was defined as the recommendation for or the receipt of HPV vaccination as recorded in the medical record or in a vaccine registry, reported by healthcare providers, parents, caregivers, or youth themselves. We recorded both initiation of vaccination, that is, the administration of the first HPV dose, as well as the up-to-date or completed age-dependent dosage (two- or three-doses). We defined HPV vaccine hesitancy using the World Health Organization definition of “the reluctance or refusal to vaccinate despite the availability of vaccines” [51].

Rurality has been defined differently by the primary studies included in this review. Rurality is defined by the US Census as a population, housing, or territory not in an urban area, with <2500 residents [13], and by the OMB by Rural–Urban Commuting Area Codes (RUCAs), with the most rural rated at 8–10 [14].

The inclusion criteria included articles published between 2010 and 2023, in the English language, and empirical research. We excluded studies that were not empirical, as well as reviews, commentaries, abstracts, and theses. Unlike systematic reviews, as yet, there are no generally accepted critical methodologic quality appraisal or risk of bias tools for scoping reviews. Our research teams rigorously followed a research protocol, with multiple internal reviews to maintain consistency, however, as inconsistency is a major concern for scoping reviews [47].

We used either the Distillr or Covidence software for the screening process, allowing for blinding. A team of 12 well-trained and closely supervised undergraduate and graduate students who were selected for a mentored research experience, led by SSG, conducted a review of abstracts. A full text review was conducted by another team of well-trained and closely supervised student researchers, again led by SSG. Any discrepancies in screening among the team members were discussed together for final inclusion.

### 2.3. Data Extraction

We extracted data using a standardized coding form adapted from Peterson et al. (2020) [10]. We collected descriptive data on each study, including publication year, study design, sample size, and participant type (e.g., children, youth, and parents). We collected the rates of HPV vaccine recommendation or uptake, and the findings on the associations among knowledge, attitudes/beliefs, and HPV vaccination. We also collected meta data about the multilevel focus of the study, that is, whether the study focused on individual children/youth, parents, healthcare providers/teams, clinics, communities, or policies. Each study was systematically coded by two independent members of the research team. Discrepancies in data extraction were resolved in discussion by a third reviewer.

### 2.4. Data Analysis

We summarized the key study characteristics using descriptive statistics (percentage, median, and mode). We conducted a narrative synthesis of the principal findings regarding knowledge, attitudes/beliefs, and HPV vaccination behaviors. The outcomes were initiation and completion of the HPV vaccination.

## 3. Results

### 3.1. Study Descriptions

We systematically reviewed 101 studies [26,27,36,37,38,52,53,54,55,56,57,58,59,60,61,62,63,64,65,66,67,68,69,70,71,72,73,74,75,76,77,78,79,80,81,82,83,84,85,86,87,88,89,90,91,92,93,94,95,96,97,98,99,100,101,102,103,104,105,106,107,108,109,110,111,112,113,114,115,116,117,118,119,120,121,122,123,124,125,126,127,128,129,130,131,132,133,134,135,136,137,138,139,140,141,142,143,144,145,146,147,148] (see Appendix B). Following the PRISMA guidelines, from 1657 abstracts retrieved, after thorough systematic evaluation by two coders using a standardized coding form, we reviewed and coded 101 papers (see Figure 1). The median year of publication was 2021; the most studies were published in 2022, about 16 years after the introduction of the HPV vaccine to the US (see Table 1). Nearly half of all studies included cross-sectional studies, with surveys in the main. Secondary analyses were the second-most common design, including data from the National Immunization Survey-Teens (NIS-T) and the 2012 Youth Risk Behavior Surveillance System (YRBSS) surveys, at 11% of all studies. Cohort studies, randomized controlled trials, and quasi-experiments were next in frequency (6.9%, each). Qualitative studies were few in number (at 6%, or six studies), as were observational (4%), pilot/feasibility studies (3%), retrospective chart reviews, and other designs (e.g., mixed methods) at 2% each.

As would be expected, children and youth, aged 17 and under, were the most common study participants (30%), followed by those aged 18–26 (26%). Parents (18.8%) and healthcare providers (14.9%) were less frequent study participants. Clinics were infrequent study participants (5%). Other participants, including community stakeholders, were more frequent participants than clinics, but were least frequent overall (11.9%). The median sample size was 101–500, although studies ranged from <50 to >10,000 for studies relying on entire populations and registry data.

Nineteen percent of the US is classified as rural, according to the US Census. Nationally, 50% of rural residents live in the southern US [149]; the South was the most frequent geographic site for study participants as well (41.6% of all studies), followed by the Midwest (20.8% of all studies). National studies were 18% of the total, followed by the West (16.8%); least frequent were studies of more than one region of the country (2%). Interestingly, while the US Census notes that 61.6% of the rural US population lives in two Northeastern states (Maine and Vermont), only one study was recruited from the Northeast (the state of New Hampshire).

### 3.2. The Characteristics of HPV Vaccine Interventions

Twenty papers studied an intervention (see Table 2). One paper tested the feasibility of an intervention [77], and one described study implementation [21]. The remaining studies tested an intervention to increase HPV vaccine uptake. The purposes of these studies varied considerably, from parental and patient education to provider education and training, to community awareness education, and general community awareness campaigns using medias and vaccine vouchers. The “other” study explored enrolling a community pharmacy in the Vaccines For Children (VFC) program. Most studies did not include a free vaccination as a part of the intervention design, although the VFC program covers most children through 18 years of age, who are as follows: Medicaid-eligible, uninsured, American Indian or Alaska Native, or underinsured [150]. Of those seven studies that provided free HPV vaccinations, two only included these if the participants were VFC-eligible. Interventions ranged from half a year to more than one year, with most being of longer duration. Within the 12 studies relying on education and training sessions that specified the length, most were one hour. Most studies that specified a session length held one session, followed in frequency by two to four sessions; five or more sessions were least frequent. The primary intervention interaction mode was in-person, followed by virtual or remote, then indirect (including social marketing campaigns). Virtual sessions (e.g., [151]) were more common than in-person during the COVID-19 pandemic, as would be expected. Two studies did not specify the number of sessions. Research teams were the primary intervention agents, followed by community medical professionals, or others (such as a *radionovela*). Only two studies relied on self-directed interventions.

### 3.3. Theoretical Models

Few studies rested on theories or models of change; most were atheoretical (see Table 3). One of the two most common models of change was the Theory of Planned Behavior [151] (TPB; undergirding six studies) with intention as a primary explanatory construct. The Health Belief Model [152] was equally frequent, underpinning eight studies, followed by the Social Ecological Model [153], undergirding three studies. The remaining models and theories each founded one study, including the Community-Based Participatory Research Model [154], the Andersen Model [155], the Shared Treatment Decision-Making Model [156], the Competing Demands Model [157], the Comprehensive Participatory Planning [158], the Consolidated Framework for Implementation Research (CFIR) [159], the Ecological Systems Theory [160], Concept Mapping [161], the Precede-Proceed Model, and the Positive Deviance Framework [162].

### 3.4. Multilevel Interventions and Change

Looking at the intervention studies from the perspective of multilevel change, we did not identify any studies at the policy level that met the selection criteria (see Table 4). Only three studies systematically assessed multilevel outcomes, that is, outcomes at two levels of change or more. One study showed an increase in initiation and completion [78], one showed a decrease in missed appointments [89], and one showed no change in the measured outcome—initiation [109] (see Table 4). At the community level of change, across five studies, the intervention increased HPV vaccine initiation and completion in four studies [73,80,116,117]. One feasibility study explored enrolling a community pharmacy as a VFC provider; the approach increased HPV vaccination initiation in Alabama [77]. None of the three studies at the clinic or system level reported a change in HPV uptake [59,70,85]. Across most previous studies, provider recommendation was cited as influential on HPV uptake; similarly, the two studies of this level of change reported an increase in initiation via systematic provider-based interventions [66,88]. Individual-level interventions were most frequent, relative to provider, clinic-, or community-level interventions (see Table 4). Four separate studies focused on the parent level of change reported increases in HPV vaccine uptake, as well as intention to vaccinate and awareness and knowledge [59,61,90,143]. Of the three studies focused on change at the young adult level, one intervention focused on both providers and young adults through a documentary film and provider education [88]. One intervention study focusing on young adults reported increased HPV vaccine series completion [135]. There was no difference between rural young adults and others in HPV vaccine uptake [27]. The three separate studies at the level of the child reported increased HPV vaccine uptake; two of these also focused on the community level via schools and educational programs [116,117,145].

### 3.5. HPV Vaccination Outcomes of Initiation, Completion, or Both

Looking at Table 5, no changes were observed in HPV vaccine initiation across studies of provider conversation training, a cancer control survivor program, a “1-2-3 Pap” informational video, or an HPV documentary movie relative to a control condition. Similarly, a web-based electronic record-linked Clinician Decision Support with or without shared decision-making tools (SDMTs) did not increase HPV vaccine initiation.

Health campaigns improved completion of the HPV vaccine in medically underserved rural colleges. In rural Iowa VFC clinics, the most frequently implemented HPV vaccine interventions engaged changing provider and patient knowledge.

Rural clinics with higher HPV vaccine up-to-date rates relative to other clinics implemented standardized workflows to identify patients due for the vaccine and had vaccine administration protocols. They had a vaccine champion. Providers administered immunizations regardless of visit type; they used clear and persuasive language to recommend or educate parents and youth.

HPV vaccination coverage was not statistically significantly different among cancer survivors participating in a Childhood Cancer Survivor Program relative to others.

The COVID-19 pandemic had a negative impact on HPV vaccination initiation and completion [36,37,38,89,96]. This finding is interpreted with caution, however, as the period of observation was not lengthy.

### 3.6. Predictors of HPV Vaccine Initiation

Reviewing Table 6, more than two-thirds of Americans had heard of HPV and the HPV vaccine, with awareness being less in rural areas than urban areas, and less among black adult women in Alabama than others. Similarly, awareness of HPV was lower among women in rural colleges than in urban ones. Males were less likely to vaccinate than women. Among rural parents, increased initiation was associated with political affiliation and not affiliating with the Baptist religion relative to Baptists. Adolescents were less likely to initiate and complete the HPV vaccine if they were not up-to-date on the hepatitis A, meningococcal, or Tdap vaccinations. Attitudes/Beliefs were the strongest predictor of mothers’ intentions to vaccinate. Social connections, including social norms and social groups, enhanced uptake; marriage diminished the intention to vaccinate.

Looking at interventions with rural residents, implementing a video community education film increased HPV knowledge gained and attitudes towards the HPV vaccine among rural adult parents, students, and providers. Providing messages through texting on cell phones promoted HPV vaccination in rural middle school students.

Among healthcare providers, rural providers were less likely to have evening/weekend hours for adolescent vaccination appointments, to have had prior experience with adolescent vaccine quality improvement projects, and to routinely recommended HPV vaccine during urgent/acute care visits than urban providers. By contrast, significantly more rural providers had standing orders to administer all recommended adolescent vaccines and reported giving HPV vaccine information to their patients/families before it was due. Collaborative communication between providers and patients was less common among rural patients than urban patients. Medical providers, rather than others in the practice, provided more favorable recommendations for the HPV vaccine.

Rural stakeholders identified education and provider influence as key to HPV vaccination; those rated as most feasible were education and coordinated/consistent messaging. Stakeholders in the Carolinas strongly supported school-based programs and approaches to strengthen confidence and demand for the HPV vaccination [79].

## 4. Discussion

### 4.1. Summary of the Results

This scoping review has systematically examined the multilevel influences on HPV vaccine uptake in rural areas across the US. The study adds to the limited number of recent reviews on rural HPV vaccine uptake in the US. Over the past 13 years, studies measured HPV vaccine initiation (at least one injection), or completion (among those who had initiated vaccination), or both. Across all types of HPV vaccine uptake, non-rural residence, female gender, and provider recommendations tended to increase vaccination, as did educational interventions, although rigorous studies of interventions were relatively few. The Vaccines For Children program increased both HPV vaccine initiation and vaccine completion, as did other forms of insurance coverage. Within each vaccination behavior, however, different factors, such as area-based poverty and sexual history, differentially influenced initiation, completion, or both.

Across the 101 studies, most increases in initiation of the HPV vaccine were among those aged 11–17. Among studies of the completion of the HPV vaccine, increased vaccination was reported among women who felt a sense of control over vaccination, and within school settings. A video educational intervention increased completion of the vaccine series. Overall, boys were less likely to be vaccinated than girls; yet, boys in high-poverty areas were more likely to complete the vaccine than comparable others.

While a multilevel framework undergirded the review, we found only 20% of intervention studies measured change at the individual, provider, clinic, or community levels of change, with the largest number of studies assessing individual-level outcomes. Even though several of the reviewed papers addressed policy-level influences, we found no policy-level interventions in rural communities.

### 4.2. Sociodemographic Influences

Some studies in this scoping review found initiation higher among those aged 11–17, yet some recent individual studies have reported strong acceptance of vaccines for those starting at age 9, and increased efficacy of those vaccines among younger children [137,163]. Of late, campaigns by the ACS, CDC, and local rural health departments, as well as the HPV Roundtable action guides, have focused on initiating the vaccine among younger children, starting at age 9. Adolescent visits to healthcare providers are less frequent than children’s visits, and vaccines are not generally a part of the visit as youth age [164]. To desexualize the vaccine, that is, detach it from decisions about engaging in sexual activity that often arise in adolescence, introducing the vaccine when children are aged 9 has been found through recent preliminary studies to increase uptake, and seems well accepted by parents [165,166,167].

The recommendations for males have traditionally lagged behind the female vaccine recommendations, resulting in lower adolescent and young adult male initiation rates [74,168,169,170,171]. Of late, however, coverage has increased dramatically among males and is now relatively comparable to that of females overall. Among rural males, however, overall, the vaccination rates still lag behind those for females. As a result, males may need different strategies for engagement than females.

The studies reviewed herein showed varied influences of areas of rural poverty. Area-based poverty was not linked with initiation, but was linked to HPV vaccine completion. A more recent individual study found a consistent influence of rural social deprivation on HPV vaccine uptake [172]. Interestingly, boys in high-poverty areas were more likely to complete the vaccine than comparable others, perhaps due to the wide reach of the VFC program.

### 4.3. Healthcare Provider Influences

One influential study in this review, followed by two more recent papers, has found that physicians who offer a presumptive announcement, stating that the vaccination would be given at that visit, increase vaccine uptake [173,174]. Strong evidence from a randomized clinical trial on presumptive announcement vs. conversational approaches in this scoping review supports the influence of the former on increased HPV vaccine uptake across rural and urban settings [175]. A recent pragmatic trial found that the approach could be effectively implemented in ongoing practice using quality improvement in diverse health department areas [173]. Recent qualitative studies of this approach in rural settings only suggest that physicians modify this approach for their rural patients, who, valuing self-sufficiency, respond less favorably to proscriptions [174]. Prescriptions could erode trust in the relationship with the physician over time [174].

### 4.4. Multilevel Observations and Interventions

However, none of the studies reviewed assessed care from all potential intervention levels—policy/community, organization/health system, provider/provider teams, to the family, and individual patients; most of the reviewed studies used individual-level interventions and outcomes only. The few system-level interventions that were studied need to be replicated to determine whether they are feasible and scalable, while yielding similar outcomes. There is promise for multilevel interventions, however, as found in a comprehensive umbrella review that included studies of both rural and urban participants [45].

Overall, the results from many large randomized clinical trials, community-based intervention trials, case–control studies, and observational studies suggest that clinically meaningful changes in cancer risk factors, cancer-related morbidity, and mortality are possible over time [176,177]. However, these approaches are often stronger in design than in implementation or evaluation [138], and more rigorous research focused on marginalized populations, including rural residents, is necessary [178].

### 4.5. Theory as a Guide

Few studies rested on theories or models of change; most were atheoretical. Yet, behavioral medicine offers a rich set of theoretical models and frameworks with which to understand multilevel influences on HPV vaccine uptake that could enrich understandings of current effective interventions and maximize their usefulness in practice. Individual, cognitive-based models such as the Theory of Planned Behavior [151], Health Belief Model [30,152] and the Theory of Reasoned Action emphasizes the importance of beliefs and values of outcomes as key variables that predict whether individuals engage in desired cancer prevention and control behaviors. The Social Ecological Model [153] emphasizes the role of the multilevel environment on HPV vaccine behavior change. Implementation Science theories and models, such as CFIR, could describe for whom theoretical models work, when they work, and when they do not (NIH, personal communication, 9/8/25). Implementation science approaches could better specify how regional cultural, economic, and healthcare system differences might influence intervention effectiveness. Implementation science approaches could subsequently inform future public health interventions, particularly to expand intervention reach.

Interventions that emphasized cancer prevention, rather than STI prevention, have been found to be more effective in promoting HPV vaccination [179,180]. In particular, within the Health Belief Model, when text messages framed the daughter’s susceptibility to HPV as a risk factor and emphasized the caregiver’s role as a protector, motivation for vaccination increased [181].

### 4.6. Limitations

As with all reviews, the quality of the scoping review is dependent upon the quality of the individual studies [46,182]. Limitations in sample sizes, geographic scope, variations in outcome measures, and the heterogeneity of the original studies on which the scoping review relies influence the rigor of the scoping review. There were variations in definitions of rurality. The scoping review does not include information for interventions or epidemiological associations that have not been examined in the included studies. In particular, we only evaluated a small number of studies of multilevel influences, as few of these studies have been conducted, so the findings may be limited.

While the protocol was not prospectively registered, it detailed the review’s inclusion and exclusion criteria and identified which and how data would be (and were) extracted and presented. This is a recommendation of JBI, a group that publishes influential guides for evidence synthesis [47].

## 5. Conclusions

This scoping review has systematically examined the multilevel influences on HPV vaccine uptake in rural areas across the US. It expands and updates the contribution of the one previous systematic review of this topic. Across all types of HPV vaccine uptake, non-rural residence, female gender, and provider recommendations tended to increase vaccination, as did educational interventions, although rigorous studies of interventions were relatively few. The Vaccines For Children program increased both HPV vaccine initiation and vaccine completion, as did other forms of insurance coverage. Adolescents themselves were the most common foci of change.

## 6. Next Steps

The findings suggest several strategies to increase rural HPV vaccine uptake [176]. Within clinics, integrating HPV vaccine surveillance data with the electronic health record could facilitate more rapid retrieval of vaccination status. The State of Michigan, for example, maintains a robust system of monitoring HPV vaccinations, but the data are not necessarily within clinical electronic health records or on dashboards, so they must be retrieved by request. Once available, routine audit and feedback of these data could increase their use by clinical teams. Further, additional workflow mapping for the HPV vaccine could facilitate routine vaccination, as noted in a previous review [40].

Among primary care physicians, training in announcing the HPV vaccine, thus integrating it with the other adolescent vaccines, could decrease hesitancy [66,183]. Practice facilitation and academic detailing with clinicians, front office staff, and others could facilitate redesign for HPV vaccination [184,185,186,187,188,189,190,191]. This redesign could include identifying community-based resources like community pharmacies that are enrolled in the Vaccines for Children program [77,192,193,194] that could increase access in rural areas. This is a critical policy-level intervention.

Importantly, tailored interventions to rural parents/caregivers could increase awareness and knowledge about the vaccine. Developing local champions among both primary care providers and rural youth for the HPV vaccine could enhance decision-making skills, disseminate accurate and unbiased information, and increase trust in the vaccine [191].

These findings will lead to approaches for vaccines to reach those who are most vulnerable to the diseases that they prevent.

## Figures and Tables

**Table 1:** Sociodemographic characteristics of those included.

	Percentage/(Number) of Studies (N = 101)	References
*Study Type*		
Cross-sectional Survey	45.5 (46)	[26,36,53,55,56,57,58,60,62,63,64,65,67,68,69,71,72,74,75,81,84,86,91,92,95,96,97,98,99,101,104,105,106,107,110,117,118,122,123,124,125,129,132,133,139]
Secondary analysis	10.9 (11)	[87,100,103,112,127,128,138,141,142,144,146]
Cohort study	6.9 (7)	[94,114,115,119,121,130,145]
Controlled trial	6.9 (7)	[59,66,76,85,90,109,135]
Mixed methods	6.9 (7)	[61,83,88,102,140,148]
Quasi-experimental	6.9 (7)	[27,70,73,89,108,116,143]
Qualitative study	5.9 (6)	[38,78,79,93,120,134]
Observational	4 (4)	[54,80,137,147]
Pilot/Feasibility study	3 (3)	[77,131,136]
Retrospective chart review	2 (2)	[52,126]
Other	2 (2)	[111,113]
*Participant Types* *		
Healthcare providers	14.9 (15)	[36,56,58,65,71,73,75,82,93,104,105,106,109,129,136]
Children and adolescents (17 and under)	28.7 (29)	[52,72,76,80,87,88,89,94,102,103,107,111,112,114,115,116,117,119,121,127,128,130,137,138,139,141,142,144,145]
Adults (18–26)	25.7 (26)	[27,53,60,62,67,68,69,74,80,85,89,95,97,98,108,110,113,122,123,124,125,126,130,135,140,147]
Parents of age-eligible children and youth	18.8 (19)	[26,55,59,63,64,73,78,89,90,91,96,99,100,101,109,131,132,133,143]
Clinics	5 (5)	[57,66,83,86,92]
Other	11.9 (12)	[38,54,61,70,77,79,84,118,120,134,146,148]
*Year of publication*		
Median year of publication 2021		
Modal publication year 2022		
*Sample size*		
<50	14.9 (15)	[38,59,70,72,77,78,79,83,85,92,93,120,134,136,143]
50–100	5.9 (6)	[58,63,90,91,145,148]
101–500	32.7 (33)	[54,55,56,57,65,67,68,69,71,73,75,76,82,84,88,89,95,96,97,102,104,108,109,113,118,123,126,129,131,133,135,140,147]
501–1000	10.9 (11)	[27,53,60,64,80,81,84,122,124,130,132]
1001–5000	12.9 (13)	[26,36,62,99,100,101,105,106,110,114,116,128,146]
5001–10,000	2 (2)	[52,61]
>10,000	20.8 (21)	[66,74,87,94,98,103,107,111,112,115,117,119,121,125,127,137,138,139,140,142,144]
*Geographic region*		
Northeast	1 (1)	[126]
Midwest	20.8 (21)	[26,38,52,53,55,56,57,64,67,81,82,85,94,97,109,119,120,121,128,129,130]
South	41.6 (42)	[27,36,59,61,63,66,68,69,70,72,73,75,77,78,79,80,88,92,93,95,99,100,104,105,106,107,108,113,116,117,123,131,132,133,134,135,136,142,143,145,146,148]
West	16.8 (17)	[58,71,76,83,84,86,89,90,91,102,103,111,114,115,140,141,147]
More than one region	2 (2)	[54,124]
National	17.8 (18)	[60,62,65,74,87,96,98,101,110,112,118,122,125,127,137,138,139,144]

* Multiple participant types: each was counted within its category, and percentages were calculated using N = 101.

**Table 2:** Characteristics of HPV vaccine intervention studies (N = 20).

Characteristic	Percentage of Studies	Study References
*Study Type*		
Feasibility	5	[77]
Intervention	90	[27,59,61,66,73,76,80,85,89,90,108,109,116,117,135,143,145]
Implementation	5	[70]
*Study Purpose* *		
Parental Education	20	[59,76,109,143]
Patient Education	20	[61,70,135,145]
Vaccine Provider Education	25	[66,76,85,109]
Community education/awareness	25	[80,88,108,116,117]
Vaccine Voucher	5	[27]
HPV Awareness/Media Campaign	10	[73,90]
Other	5	[77]
*Included Free Vaccination*		
Yes	25	[27,61,116,117,135]
VFC-eligible only	10	[77,80]
No	65	[59,66,70,73,76,85,88,89,90,108,109,141,145]
*Intervention Duration* **		
<0.5 year	30	[59,66,73,90,143,145]
0.5 year–1 year	20	[70,77,80,135]
>1 year	45	[30,61,76,85,89,108,109,116,117]
Unspecified	5	[88]
*Duration of Training and Education* *Sessions (N = 12)*		
Under 1 h	17	[135,143]
1 h	50	[66,76,80,89,109,145]
Unspecified	33	[85,108,116,117]
*Number of Sessions/Activities (N = 14)*		
One	36	[66,80,90,109,135]
Two to four	29	[76,85,89,143]
Five or more	21	[116,117,145]
Unspecified	14	[90,110]
*Mode of Interaction* *		
Indirect	20	[73,76,89,109]
In-person	65	[27,59,61,66,76,77,85,88,90,108,116,117,143]
Virtual or remote	25	[77,80,85,88,145]
Unspecified	10	[70,135]
*Intervener* *		
Research team	50	[27,73,76,77,85,88,89,109]
Self-directed	10	[70,135]
Community medical professionals	25	[59,80,116,117,143]
Other	25	[61,66,80,90,145]

* Will not equal 100%; ** Time included intervention, control period, and evaluation period of studies.

**Table 3:** Theories and models of change (N = 35).

Theory/Model/Framework	N	Study References
Anderson Model	1	[69]
Shared Treatment Decision-Making Model	1	[101]
Community-Based Participatory Research (CBPR)	1	[148]
Competing Demands Model	1	[65]
Comprehensive Participatory Planning	1	[148]
Consolidated Framework for Implementation Research (CFIR)	1	[38]
Ecological Systems Theory	1	[99]
Evidence-Based Practice Model	1	[59]
Extended Parallel Process Model	1	[109]
Health Belief Model	7	[71,72,73,108,109,132,133]
Concept Mapping	1	[54]
Organizational Developmental Theory	1	[109]
Positive Deviance Framework	1	[83]
Precede-Proceed Model	1	[134]
Social Ecological Framework	3	[79,91,134]
Theory of Planned Behavior	7	[55,56,67,68,118,123,135]
Theory of Reasoned Action	1	[109]
Vaccine Perceptions, Accountability and Adherence Model	1	[102]

**Table 4:** Multilevel intervention findings (N = 20).

Intervention Level	Outcome Trend *
Multilevel	
	Increase
	Initiation and Completion [76]
	No Change
	Initiation Only [109]
	Decrease
	Missed Opportunities [89]
Community-level	
	Increase
	Completion and Initiation [80,116,117]
	Initiation [73,77] Attitudes toward, knowledge of HPV vaccination [108]
Clinic-level	
	No Change
	Completion Only [85] Initiation or Completion [59,70]
Provider level/Team level	
	Increase
	Initiation Only [66,88]
Parent/Adult-level	
	Increase
	Completion and Initiation [61]
	Initiation Only [59]
	Intention to Vaccinate [143] Awareness and knowledge [90]
Individual Young Adult	No difference (rural women only)
	Initiation [27,88] Increase Completion [135]
Individual Child/Youth	Increase
	Initiation [145]
	Completion and Initiation [116,117]

* Studies listed multiple times had multiple primary findings with different trends in vaccination outcomes. Other denotes that either the data were collected cross-sectionally, or a trend could not be detected from the data provided.

**Table 5:** HPV vaccination uptake by initiation and completion.

*Initiation only: Increase*	
	**Multilevel Intervention** Significantly more multilevel intervention participants received the vaccine at 3 months than the comparison participants [109]. **Vouchers for Free Vaccines, Social Marketing Campaigns** Less than 50% of eligible individuals redeemed the voucher to receive dose one of the HPV vaccine for free in rural Appalachia [28].
	A social marketing campaign initiated by county health departments in a primarily rural and a racially diverse part of North Carolina increased HPV vaccine uptake among preteen girls for whom the vaccine is routinely recommended [73]. **Provider Recommendation** Six-month increases in HPV vaccination coverage were larger for patients in clinics that received provider-based announcement training versus those in control clinics (5.4% difference, 95% confidence interval: 1.1–9.7%) [66]. In the NIS-Teen 2017 data, receiving a medical provider recommendation was significantly associated with series initiation [102] Provider recommendation that the HPV vaccine was significantly associated with the child being vaccinated that day, as well as scheduling vaccination in Alabama rural clinics. Parents who got the impression that “there was no hurry” were less likely to vaccinate their child that day [63]. Pharmacist-delivered educational presentation increased intention to vaccinate according to guidelines from 35% (N = 12) to 44% (N = 15) [143]. **Individuals Engaging in Protective Behaviors** Among Appalachian women, those engaging in behaviors that increase their risk for HPV infection were more likely to refuse the vaccine. Those women engaging in protective health behaviors were more likely to accept the vaccine [24].
*Initiation: No change*	
	Provider conversation training did not differ from control clinics [66]
*Completion only: Increase*	
	Health campaigns to increase the HPV vaccine in rural and medically underserved college campuses need to target both genders to complete the vaccination process [67]. In rural Iowa VFC clinics, commonly implemented interventions focused on provider knowledge and patient education. Least commonly implemented interventions required systematic changes, such as reminder/recall and follow-up after missed appointments [57].
*Initiation and Completion: Increase*	
	Rural clinics with higher HPV vaccine up-to-date rates differed from those with lower rates as they implemented standardized workflows to identify patients due for the vaccine and had vaccine administration protocols; they had vaccine champions. They provided immunizations regardless of visit type; clear, persuasive language to recommend or educate parents and patients [83].
*Initiation and Completion: No Change*	
	HPV vaccination coverage was not statistically significantly different among CCSP patients (60.0%) compared to controls (66.3%). The proportions receiving 2 doses (CCSP patients 21.5% vs. controls 20.7%) and 3 doses (28.5% vs. 30.1%) were comparable between CCSP patients and controls [130].
*Initiation and Completion: Decrease*	
	The COVID-19 pandemic had a negative impact on HPV vaccination [37].

MSA: Metropolitan Statistical Area; LHD: local health department; HPSA: health provider shortage areas; PBC: perceived behavioral control; RUCA: Rural–Urban Commuting Area Codes; CCSP: childhood cancer survivor program; GNC: Gender nonconforming; GNB: Gender nonbinary; OTM: other healthcare team members (not physicians, PAs, NPs, or residents); HCPs: Healthcare providers; SAAB: sex assigned at birth; CDS: clinical decision support; SDMT: shared decision-making tools; UC: usual care.

**Table 6:** Summary of the predictors of HPV vaccine Initiation.

*Awareness of HPV*	
	In 2013, 68% of all Americans had heard of HPV and the HPV vaccine. Those in rural areas were less likely than those in urban areas to know that HPV causes cervical cancer [62]. Slightly more than half of the black participants in Alabama were aware of HPV (62.5%) and the HPV vaccine (62.1%). Marriage or partnership lowered awareness; family cancer history, self-reported health status, employment, and participation in social groups increased awareness [95].
*Attitudes/Beliefs, Intentions to vaccinate, subjective norms*	
	Significant initial uptake difference between urban and rural college women; rural clinic women are less likely to follow up [26]. Attitudes were the strongest predictor of mothers’ intentions to vaccinate [95], but intentions were not high [55]. Subjective norms also influence intention [55].
*Political Affiliation and Religion*	
	Increase in initiation associated with political affiliation (Democratic affiliation). Political affiliation explained most of the variation in vaccine confidence and intention/uptake between rural and other respondents [96]. Non-Baptists were 3.6 times more likely to vaccinate than Baptists [133].
*Up-to-date with other vaccinations*	
	Adolescents were less likely to initiate and complete the HPV vaccine if they were not up-to-date on the hepatitis A, meningococcal, or Tdap vaccinations [52].
*Gender*	
	Men aged 40 and younger were less likely to have any human papillomavirus vaccination than women [81].
*Text messaging and DVD educational programs*	
	Providing messages through texting on cell phones could promote the HPV vaccine in rural middle school students [72]. DVD community education film, “Someone You Love: The HPV Epidemic,” increased HPV knowledge gained and attitudes towards the HPV vaccine in rural Virginia locations [108].
*Provider access, influence, collaborative communication, and HPV education*	
	Relative to urban midwestern providers, significantly fewer rural providers had evening/weekend adolescent vaccination appointments available, had prior experience with adolescent vaccine quality improvement projects, and routinely recommended the HPV vaccine during urgent/acute care visits. Significantly more rural providers had standing orders to administer all recommended adolescent vaccines, and reported giving the HPV vaccine information to their patients/families before it was due [82]. Pediatricians in the western US reported a higher number of challenges limiting HPV vaccination, higher HPV vaccination knowledge, and more favorable HPV vaccination recommendation practices compared to other healthcare team members (OTM, including nurses, medical assistants clinic staff, administrators, and stakeholders (like community health workers) [58]. Collaborative communication affected urban–rural uptake disparity; poorer, less educated, and rural parents reported less communication [56]. Collaborative communication between providers and patients is less common among rural residents, and may account for differences—and lack of differences—in HPV vaccination among some subgroups of adolescent girls [101]. Clusters rated as most important by rural stakeholders included: education and provider influence; those rated as most feasible were education and coordinated/consistent messaging [54].
*School-based programs*	
	Stakeholders in the Carolinas strongly supported school-based programs and approaches to strengthen confidence and demand for HPV vaccination [79].

## Data Availability

The data supporting the reported results are published.

## Appendix A

### Appendix A.1. Search Strategy

The bibliographic database searches were run from 2010 to 2023; the search conducted at the start of 2024 captured any articles published at the end of 2023. The searches were conducted by an expert informaticist on the following dates [195]:

9 January 2024

6 July 2023

16 June 2021

**The following bibliographic databases were searched:**

Medline (OVID)

Embase (Elsevier)

CINAHL (EBSCOhost)

PsycInfo (EBSCOhost)

Cochrane (Wiley)

Sociological Abstracts (ProQuest)

Scopus (Elsevier)

#### Appendix A.1.1. Medline

1. exp Papillomavirus Infections/or exp Alphapapillomavirus/or (“papillomavirus infection*” or “Human Papillomavirus” or “Human papilloma virus” or HPV).tw,kw.
2. Exp immunization/or exp Immunization programs/or exp Papillomavirus Vaccines/or Vaccines/or (vaccin* OR immuniz* OR immunis* OR inoculat* OR Nine-valent OR “nine valent” OR bivalent OR quadrivalent OR Gardasil OR cervarix).tw,kw.
3. Exp patient acceptance of health care/OR exp Health Knowledge, Attitudes, Practice/OR vaccination refusal/OR anti-vaccination movement/OR exp decision making/OR trust/OR exp risk assessment OR exp religion/OR (accept* OR aware* OR attitude* OR knowledge OR belief* OR view* OR opinion* OR barrier* OR support* OR behave*OR decision OR decide OR intent* OR undecided OR hesita* OR doubt* OR refus* OR reject* OR omission* OR omit* OR object OR objection OR incomplet* OR delay* OR suboptimal* OR intent* OR know* OR perceive* OR percept* OR perspective* OR understand* OR prefer* OR risk* OR uptake* OR will* OR hesitan* OR reluctan* OR fear OR concern* OR trust OR uncertain* or distrust OR anti-vax* OR anti-vacc* OR antivax* or antivaccin* OR wary OR religion OR religious).tw,kw.
4. Exp rural health services/OR rural population/OR rural health/OR “Hospitals, Rural”/OR Medically Underserved Area/OR exp population dynamics/OR exp residence characteristics/OR (remote OR rural OR Appalachia OR (regional adj3 disparit*) OR “small town*” OR (region* adj3 disparities) OR ((geographic* OR medical*) adj3 (underserv* OR underrepresent*)) OR (underserv* adj3 (population* OR communit* OR area*)) OR (shortage adj3 area)).tw,kw.
5. (1 AND 2 AND 3 AND 4)

#### Appendix A.1.2. Embase

1. ‘Papillomavirus Infection’/exp OR ‘Alphapapillomavirus’/exp OR (“Human Papillomavirus” OR “papillomavirus infection*” OR “Human papilloma virus” OR HPV): ti, ab, kw
2. ‘immunization’/exp OR ‘wart virus Vaccine’/de OR ‘Vaccine’/de OR (vaccin* OR immuniz* OR immunis* OR inoculat* OR Nine-valent OR “nine valent” OR bivalent OR quadrivalent OR Gardasil OR cervarix): ti, ab, kw
3. ‘Patient attitude’/exp OR ‘attitude to health’/de OR ‘anti-vaccination movement’/de OR ‘patient decision making’/de OR ‘trust’/de OR ‘risk assessment’/de OR ‘religion’/exp OR (accept* OR aware* OR attitude* OR knowledge OR belief* OR view* OR opinion* OR barrier* OR support* OR behave* OR decision OR decide OR intent* OR undecided OR hesita* OR doubt* OR refus* OR reject* OR omission* OR omit* OR object OR objection OR incomplet* OR delay* OR suboptimal* OR intent* OR know* OR perceive* OR percept* OR perspective* OR understand* OR prefer* OR risk* OR uptake* OR will* OR hesitan* OR reluctan* OR fear OR concern* OR trust OR uncertain* OR distrust OR anti-vax* OR anti-vacc* OR antivax* OR antivaccin* OR wary OR religion OR religious): ti, ab, kw
4. ‘rural health care’/exp OR ‘rural population’/de OR ‘rural health’/de OR ‘rural hospital’/de OR ‘Medically Underserved’/de OR ‘population dynamics’/exp OR ‘demography”/de OR (remote OR rural OR Appalachia OR (regional NEAR/3 disparit*) OR “small town*” OR (region* NEAR/3 disparities) OR ((geographic* OR medical*) NEAR/3 (underserv* OR underrepresent*)) OR (underserv* NEAR/3 (population* OR communit* OR area*)) OR (shortage NEAR/3 area)): ti, ab, kw

#5:
[06-07-2023]/sd
#6:
[embase]/lim NOT ([embase]/lim AND [medline]/lim)
#7.
(#1 AND #2 AND #3 AND #4 AND #5 AND #6)

#### Appendix A.1.3. CINAHL

1. (MH “Papillomavirus Infections+”) OR (TI “papillomavirus infection*” OR AB “papillomavirus infection*”) OR (TI “Human Papillomavirus” OR AB “Human Papillomavirus”) OR (TI “Human papilloma virus” OR AB “Human papilloma virus”) OR (TI HPV OR AB HPV)
2. (MH “immunization”) OR (MH “Papillomavirus Vaccines”) OR (MH “Viral Vaccines”) OR ((TI vaccin* OR AB vaccin*) OR (TI immuniz* OR AB immuniz*) OR (TI immunis* OR AB immunis*) OR (TI inoculat* OR AB inoculat*) OR (TI Nine-valent OR AB Nine-valent) OR (TI “nine valent” OR AB “nine valent”) OR (TI bivalent OR AB bivalent) OR (TI quadrivalent OR AB quadrivalent) OR (TI Gardasil OR AB Gardasil) OR (TI cervarix OR AB cervarix)
3. (MH “Patient Attitudes”) OR (MH “Attitude to Vaccines”) OR (MH “Health Knowledge”) OR (MH “anti-vaccination movement”) OR (MH “decision making, patient”) OR (MH “trust”) OR (MH “risk assessment”) OR (MH “religion and relgions+”) OR ((TI accept* OR AB accept*) OR (TI aware* OR AB aware*) OR (TI attitude* OR AB attitude*) OR (TI knowledge OR AB knowledge) OR (TI belief* OR AB belief*) OR (TI view* OR AB view*) OR (TI opinion* OR AB opinion*) OR (TI barrier* OR AB barrier*) OR (TI support* OR AB support*) OR (TI behave* OR AB behave*) OR (TI decision OR AB decision) OR (TI decide OR AB decide) OR (TI intent* OR AB intent*) OR (TI undecided OR AB undecided) OR (TI hesita* OR AB hesita*) OR (TI doubt* OR AB doubt*) OR (TI refus* OR AB refus*) OR (TI reject* OR AB reject*) OR (TI omission* OR AB omission*) OR (TI omit* OR AB omit*) OR (TI object OR AB object) OR (TI objection OR AB objection) OR (TI incomplet* OR AB incomplet*) OR (TI delay* OR AB delay*) OR (TI suboptimal* OR AB suboptimal*) OR (TI intent* OR AB intent*) OR (TI know* OR AB know*) OR (TI perceive* OR AB perceive*) OR (TI percept* OR AB percept*) OR (TI perspective* OR AB perspective*) OR (TI understand* OR AB understand*) OR (TI prefer* OR AB prefer*) OR (TI risk* OR AB risk*) OR (TI uptake* OR AB uptake*) OR (TI will* OR AB will*) OR (TI hesitan* OR AB hesitan*) OR (TI reluctan* OR AB reluctan*) OR (TI fear OR AB fear) OR (TI concern* OR AB concern*) OR (TI trust OR AB trust) OR (TI uncertain* OR AB uncertain*) OR (TI distrust OR AB distrust) OR (TI anti-vax* OR AB anti-vax*) OR (TI anti-vacc* OR AB anti-vacc*) OR (TI antivax* OR AB antivax*) OR (TI antivaccin* OR AB antivaccin*) OR (TI wary OR AB wary) OR (TI religion OR AB religion) OR (TI religious OR AB religious)
4. (MH “rural health services”) OR (MH “rural population”) OR (MH “rural health”) OR (MH “Hospitals, Rural”) OR (MH “Medically Underserved Area”) OR (MH “population characteristics”) OR (MH “residence characteristics”) OR ((TI remote OR AB remote) OR (TI rural OR AB rural) OR (TI Appalachia OR AB Appalachia) OR ((TI regional OR AB regional) N3 (TI disparit* OR AB disparit*)) OR (TI “small town*” OR AB “small town*”) OR ((TI region* OR AB region*) N3 (TI disparities OR AB disparities)) OR (((TI geographic* OR AB geographic*) OR (TI medical* OR AB medical*)) N3 ((TI underserv* OR AB underserv*) OR (TI underrepresent* OR AB underrepresent*))) OR ((TI underserv* OR AB underserv*) N3 ((TI population* OR AB population*) OR (TI communit* OR AB communit*) OR (TI area* OR AB area*))) OR ((TI shortage OR AB shortage) N3 (TI area OR AB area)))

#5:
(1 AND 2 AND 3 AND 4 AND 5)

#### Appendix A.1.4. Scopus

1. TITLE-ABS-KEY (“Papillomavirus Infection*”) OR TITLE-ABS-KEY (“papillomavirus infection*”) OR TITLE-ABS-KEY (“Alphapapillomavirus”) OR TITLE-ABS-KEY (“Human Papillomavirus”) OR TITLE-ABS-KEY (“Human papilloma virus”) OR TITLE-ABS-KEY (“HPV”)
2. TITLE-ABS-KEY (“immunization”) OR TITLE-ABS-KEY (“Vaccin*”) OR TITLE-ABS-KEY (“immuniz*”) OR TITLE-ABS-KEY (“immunis*”) OR TITLE-ABS-KEY (“inoculat*”) OR TITLE-ABS-KEY (“Nine-valent”) OR TITLE-ABS-KEY (“nine valent”) OR TITLE-ABS-KEY (“bivalent”) OR TITLE-ABS-KEY (“quadrivalent”) OR TITLE-ABS-KEY (“Gardasil”) OR TITLE-ABS-KEY (“cervarix”)
3. INDEXTERMS (“patient acceptance of health care”) OR INDEXTERMS (“Health Knowledge, Attitudes, Practice”) OR INDEXTERMS (“anti-vaccination movement”) OR INDEXTERMS (“decision making”) OR INDEXTERMS (“trust”) OR INDEXTERMS (“risk assessment”) OR TITLE-ABS-KEY (“accept*”) OR TITLE-ABS-KEY (“aware*”) OR TITLE-ABS-KEY (“attitude*”) OR TITLE-ABS-KEY (“knowledge”) OR TITLE-ABS-KEY (“belief*”) OR TITLE-ABS-KEY (“view*”) OR TITLE-ABS-KEY (“opinion*”) OR TITLE-ABS-KEY (“barrier*”) OR TITLE-ABS-KEY (“support*”) OR TITLE-ABS-KEY (“behave*”) OR TITLE-ABS-KEY (“decision”) OR TITLE-ABS-KEY (“decide”) OR TITLE-ABS-KEY (“intent*”) OR TITLE-ABS-KEY (“undecided”) OR TITLE-ABS-KEY (“hesita*”) OR TITLE-ABS-KEY (“doubt*”) OR TITLE-ABS-KEY (“refus*”) OR TITLE-ABS-KEY (“reject*”) OR TITLE-ABS-KEY (“omission*”) OR TITLE-ABS-KEY (“omit*”) OR TITLE-ABS-KEY (“object”) OR TITLE-ABS-KEY (“objection”) OR TITLE-ABS-KEY (“incomplet*”) OR TITLE-ABS-KEY (“delay*”) OR TITLE-ABS-KEY (“suboptimal*”) OR TITLE-ABS-KEY (“intent*”) OR TITLE-ABS-KEY (“know*”) OR TITLE-ABS-KEY (“perceive*”) OR TITLE-ABS-KEY (“percept*”) OR TITLE-ABS-KEY (“perspective*”) OR TITLE-ABS-KEY (“understand*”) OR TITLE-ABS-KEY (“prefer*”) OR TITLE-ABS-KEY (“risk*”) OR TITLE-ABS-KEY (“uptake*”) OR TITLE-ABS-KEY (“will*”) OR TITLE-ABS-KEY (“hesitan*”) OR TITLE-ABS-KEY (“reluctan*”) OR TITLE-ABS-KEY (“fear”) OR TITLE-ABS-KEY (“concern*”) OR TITLE-ABS-KEY (“trust”) OR TITLE-ABS-KEY (“uncertain*”) OR TITLE-ABS-KEY (“distrust”) OR TITLE-ABS-KEY (“anti-vax*”) OR TITLE-ABS-KEY (“anti-vacc*”) OR TITLE-ABS-KEY (“antivax*”) OR TITLE-ABS-KEY (“antivaccin*”) OR TITLE-ABS-KEY (“wary”) OR TITLE-ABS-KEY (“religion”) OR TITLE-ABS-KEY (“religious”)
4. INDEXTERMS (“Medically Underserved Area”) OR INDEXTERMS (“population dynamics”) OR “exp residence characteristics” OR (TITLE-ABS-KEY (“remote”) OR TITLE-ABS-KEY (“rural”) OR TITLE-ABS-KEY (“Appalachia”) OR (TITLE-ABS-KEY (“regional”) W/3 TITLE-ABS-KEY (“disparit*”)) OR TITLE-ABS-KEY (“small town*”) OR (TITLE-ABS-KEY (“region*”) W/3 TITLE-ABS-KEY (“disparities”)) OR ( (TITLE-ABS-KEY (“geographic*”) OR TITLE-ABS-KEY (“medical*”)) W/3 (TITLE-ABS-KEY (“underserv*”) OR TITLE-ABS-KEY (“underrepresent*”))) OR (TITLE-ABS-KEY (“underserv*”) W/3 (TITLE-ABS-KEY (“population*”) OR TITLE-ABS-KEY (“communit*”) OR TITLE-ABS-KEY (“area*”))) OR (TITLE-ABS-KEY (“shortage”) W/3 TITLE-ABS-KEY (“area”)))

#5:
ORIG-LOAD-DATE AFT 20230706
#5
(#1 AND #2 AND #3 AND #4 AND #5)

#### Appendix A.1.5. PsycInfo

1. (DE “Human Papillomavirus”) OR ((TI “Human Papillomavirus” OR AB “Human Papillomavirus”) OR (TI “papillomavirus infection*” OR AB “papillomavirus infection*”) OR (TI “Human papilloma virus” OR AB “Human papilloma virus”) OR (TI HPV OR AB HPV))
2. (DE “immunization”) OR ((TI vaccin* OR AB vaccin*) OR (TI immuniz* OR AB immuniz*) OR (TI immunis* OR AB immunis*) OR (TI inoculat* OR AB inoculat*) OR (TI Nine-valent OR AB Nine-valent) OR (TI “nine valent” OR AB “nine valent”) OR (TI bivalent OR AB bivalent) OR (TI quadrivalent OR AB quadrivalent) OR (TI Gardasil OR AB Gardasil) OR (TI cervarix OR AB cervarix))
3. (DE “Health Knowledge”) OR (DE “decision making”) OR (DE “trust”) OR (DE “risk assessment”) OR (DE “religion+”) OR ((TI accept* OR AB accept*) OR (TI aware* OR AB aware*) OR (TI attitude* OR AB attitude*) OR (TI knowledge OR AB knowledge) OR (TI belief* OR AB belief*) OR (TI view* OR AB view*) OR (TI opinion* OR AB opinion*) OR (TI barrier* OR AB barrier*) OR (TI support* OR AB support*) OR (TI behave* OR AB behave*) OR (TI decision OR AB decision) OR (TI decide OR AB decide) OR (TI intent* OR AB intent*) OR (TI undecided OR AB undecided) OR (TI hesita* OR AB hesita*) OR (TI doubt* OR AB doubt*) OR (TI refus* OR AB refus*) OR (TI reject* OR AB reject*) OR (TI omission* OR AB omission*) OR (TI omit* OR AB omit*) OR (TI object OR AB object) OR (TI objection OR AB objection) OR (TI incomplet* OR AB incomplet*) OR (TI delay* OR AB delay*) OR (TI suboptimal* OR AB suboptimal*) OR (TI intent* OR AB intent*) OR (TI know* OR AB know*) OR (TI perceive* OR AB perceive*) OR (TI percept* OR AB percept*) OR (TI perspective* OR AB perspective*) OR (TI understand* OR AB understand*) OR (TI prefer* OR AB prefer*) OR (TI risk* OR AB risk*) OR (TI uptake* OR AB uptake*) OR (TI will* OR AB will*) OR (TI hesitan* OR AB hesitan*) OR (TI reluctan* OR AB reluctan*) OR (TI fear OR AB fear) OR (TI concern* OR AB concern*) OR (TI trust OR AB trust) OR (TI uncertain* OR AB uncertain*) OR (TI distrust OR AB distrust) OR (TI anti-vax* OR AB anti-vax*) OR (TI anti-vacc* OR AB anti-vacc*) OR (TI antivax* OR AB antivax*) OR (TI antivaccin* OR AB antivaccin*) OR (TI wary OR AB wary) OR (TI religion OR AB religion) OR (TI religious OR AB religious))
4. (DE “rural environments”) OR (DE “rural health”) OR ((TI remote OR AB remote) OR (TI rural OR AB rural) OR (TI Appalachia OR AB Appalachia) OR ((TI regional OR AB regional) N3 (TI disparit* OR AB disparit*)) OR (TI “small town*” OR AB “small town*”) OR ((TI region* OR AB region*) N3 (TI disparities OR AB disparities)) OR (((TI geographic* OR AB geographic*) OR (TI medical* OR AB medical*)) N3 ((TI underserv* OR AB underserv*) OR (TI underrepresent* OR AB underrepresent*))) OR ((TI underserv* OR AB underserv*) N3 ((TI population* OR AB population*) OR (TI communit* OR AB communit*) OR (TI area* OR AB area*))) OR ((TI shortage OR AB shortage) N3 (TI area OR AB area)))
5. (#1 AND #2 AND #3 AND #4)

#### Appendix A.1.6. Cochrane

1. [mh “Papillomavirus Infections”] OR [mh Alphapapillomavirus] OR (“papillomavirus infection*” OR “Human Papillomavirus” OR “Human papilloma virus” OR HPV): ti, ab, kw.
2. [mh immunization] OR [mh “Immunization programs”] OR [mh “Papillomavirus Vaccines”] OR [mh Vaccines] OR (vaccin* OR immuniz* OR immunis* OR inoculat* OR Nine-valent OR “nine valent” OR bivalent OR quadrivalent OR Gardasil OR cervarix): ti, ab, kw.
3. [mh “patient acceptance of health care”] OR [mh “Health Knowledge, Attitudes, Practice”] OR [mh “vaccination refusal”] OR [mh “anti-vaccination movement”] OR [mh “decision making”] OR [mh trust] OR [mh “risk assessment OR exp religion”] OR (accept* OR aware* OR attitude* OR knowledge OR belief* OR view* OR opinion* OR barrier* OR support* OR behave* OR decision OR decide OR intent* OR undecided OR hesita* OR doubt* OR refus* OR reject* OR omission* OR omit* OR object OR objection OR incomplet* OR delay* OR suboptimal* OR intent* OR know* OR perceive* OR percept* OR perspective* OR understand* OR prefer* OR risk* OR uptake* OR will* OR hesitan* OR reluctan* OR fear OR concern* OR trust OR uncertain* OR distrust OR anti-vax* OR anti-vacc* OR antivax* OR antivaccin* OR wary OR religion OR religious): ti, ab, kw.
4. [mh “rural health services”] OR [mh “rural population”] OR [mh “rural health”] OR [mh “Hospitals, Rural”] OR [mh “Medically Underserved Area”] OR [mh “population dynamics”] OR [mh “residence characteristics”] OR (remote OR rural OR Appalachia): ti, ab, kw. OR (regional NEAR/3 disparit*): ti, ab, kw. OR (“small” NEAR/2 town*): ti, ab, kw. OR (region* NEAR/3 disparities): ti, ab, kw. OR ((geographic* OR medical*) NEAR/3 (underserv* OR underrepresent*)): ti, ab, kw. OR (underserv* NEAR/3 (population* OR communit* OR area*)): ti, ab, kw. OR (shortage NEAR/3 area): ti, ab, kw.

#### Appendix A.1.7. Sociological Abstracts

1. TI (“papillomavirus infection*” OR “Human Papillomavirus” or “Human papilloma virus” or HPV) OR AB (“papillomavirus infection*” OR “Human Papillomavirus” or “Human papilloma virus” or HPV)
2. SU (“immunization”) OR SU (“vaccination”) or TI (vaccin* OR immuniz* OR immunis* OR inoculat* OR Nine-valent OR “nine valent” OR bivalent OR quadrivalent OR Gardasil OR cervarix) OR AB (vaccin* OR immuniz* OR immunis* OR inoculat* OR Nine-valent OR “nine valent” OR bivalent OR quadrivalent OR Gardasil OR cervarix)
3. SU (“Health attitudes”) OR MAINSUBJECT.EXACT.EXPLODE (“Decision Making”) OR SU (“trust”) OR SU (“risk”) OR SU (“religions”) OR (TI (accept* OR aware* OR attitude* OR knowledge OR belief* OR view* OR opinion* OR barrier* OR support* OR behave* OR decision OR decide OR intent* OR undecided OR hesita* OR doubt* OR refus* OR reject* OR omission* OR omit* OR object OR objection OR incomplet* OR delay* OR suboptimal* OR intent* OR know* OR perceive* OR percept* OR perspective* OR understand* OR prefer* OR risk* OR uptake* OR will* OR hesitan* OR reluctan* OR fear OR concern* OR trust OR uncertain* or distrust OR anti-vax* OR anti-vacc* OR antivax* or antivaccin* OR wary OR religion OR religious) OR AB (accept* OR aware* OR attitude* OR knowledge OR belief* OR view* OR opinion* OR barrier* OR support* OR behave* OR decision OR decide OR intent* OR undecided OR hesita* OR doubt* OR refus* OR reject* OR omission* OR omit* OR object OR objection OR incomplet* OR delay* OR suboptimal* OR intent* OR know* OR perceive* OR percept* OR perspective* OR understand* OR prefer* OR risk* OR uptake* OR will* OR hesitan* OR reluctan* OR fear OR concern* OR trust OR uncertain* or distrust OR anti-vax* OR anti-vacc* OR antivax* or antivaccin* OR wary OR religion OR religious))
4. SU (“Rural Population”) OR SU (“Rural Areas”) OR SU (“Rurality”) OR SU (“Rural Communities”) OR SU (“Rural Urban Differences”) OR SU (“residence”) OR TI (remote OR rural OR Appalachia OR (regional adj3 disparit*) OR “small town*” OR (region* adj3 disparities) OR ((geographic* OR medical*) adj3 (underserv* OR underrepresent*)) OR (underserv* adj3 (population* OR communit* OR area*)) OR (shortage adj3 area)) OR AB (remote OR rural OR Appalachia OR (regional adj3 disparit*) OR “small town*” OR (region* adj3 disparities) OR ((geographic* OR medical*) adj3 (underserv* OR underrepresent*)) OR (underserv* adj3 (population* OR communit* OR area*)) OR (shortage adj3 area))
5. (1 AND 2 AND 3 AND 4)

## Appendix B. Full Study Characteristics (N = 101)

**Reference**	**Publication Year**	**Location**	**Study Years**	**Purpose/Aims**	**Study Type**	**Sample Size**	**Participant Type**	**Theory, Model, and Framework**	**Primary Outcomes Measured**	**Other Outcomes Measured**	**Primary Findings**	**Other Findings**
Bhatta MP, Phillips L. Human papillomavirus vaccine awareness, uptake, and parental and healthcare provider communication among 11- to 18-year-old adolescents in a rural Appalachian Ohio county in the United States. *J Rural Health Winter*. 2015;31(1):67–75. doi:10.1111/jrh.12079 [26]	2014	Midwest	2012	Examine the levels of adolescent HPV vaccine awareness, uptake, and parental and healthcare provider communication; assess the relationship between the parental and healthcare provider communication regarding the HPV vaccine, and the vaccine uptake from the adolescent perspective	Cross-sectional survey	1299	Parents of adolescents		HPV vaccine initiation and awareness	Parental and provider communication about HPV vaccine	49.2% of respondents reported that they have heard of the HPV vaccine. Overall, 19.4% of the adolescents indicated having a discussion with their parents about the HPV vaccine. Nearly a quarter (24.6%) of the adolescents indicated having a healthcare provider discuss the HPV vaccine with them.	Both parental and healthcare provider communication were significantly associated with HPV vaccine uptake in this population (*p* < 0.0001)
Crosby RA, Casey BR, Vanderpool R, Collins T, Moore GR. Uptake of free HPV vaccination among young women: a comparison of rural versus urban rates. *J Rural Health Winter*. 2011;27(4):380–384. doi:10.1111/j.1748-0361.2010.00354.x [27]	2011	South	2007–2009	Compare rates of initial HPV vaccine uptake, offered at no cost, between a rural clinic, a rural community college, and an urban college clinic and to identify rural–urban differences in uptake of free booster doses	quasi-experimental study	706	Adults		HPV vaccine Initiation and Completion		The contrast in initial uptake between urban clinic women and rural college women was significant (*p* < 0.0001), but the difference in initial uptake between urban clinic women and rural clinic women was not significant (*p* = 0.42). Rural clinic women were about 7 times more likely than urban clinic women (*p* < 0.0001) to not return for at least 1 follow-up dose. The difference between urban clinic women and rural college women was significant for follow-up vaccine doses (*p* = 0.014).	
* Osaghae I, Chido-Amajuoyi OG, Shete S. Healthcare Provider Recommendations and Observed Changes in HPV Vaccination Acceptance during the COVID-19 Pandemic. Vaccines. 2022;10(9):1515. doi:10.3390/vaccines10091515 [36]	2022	South	2021	Examine the association between HPV vaccination recommendation by HCPs and their observed changes in HPV Vaccination acceptance during the COVID-19 pandemic	Cross-sectional study	1283	Providers		HPV vaccine initiation		554 (77.5%) reported no change, 99 (13.9%) reported a decrease, and 62 (8.7%) reported an increase in HPV vaccination acceptance during the COVID-19 pandemic. Providers who recommended the vaccine often/always had 46% (OR = 0.54; 95%CI: 0.30–0.96) lower odds of reporting a decrease in HPV vaccination acceptance during the COVID-19 pandemic	
Ryan G, Gilbert PA, Ashida S, Charlton ME, Scherer A, Askelson NM. Challenges to Adolescent HPV Vaccination and Implementation of Evidence-Based Interventions to Promote Vaccine Uptake During the COVID-19 Pandemic: “HPV Is Probably Not at the Top of Our List.” Prev Chronic Dis. 2022;19. doi:10.5888/pcd19.210378 [38]	2022	Midwest	2020	Assess how the COVID-19 pandemic impacted opportunities for HPV vaccination delivery and EBI implementation	Qualitative	18	Clinic Managers and administrators	Consolidated Framework for Implementation Research	Clinic challenges to implementing HPV vaccines during COVID-19 pandemic		The pandemic led to an overall decrease in HPV vaccinations as well as routine care. Additionally, the pandemic disrupted EBI work (evidence-based interventions)	
Adjei Boakye E, Fedorovich Y, White M, et al. Rural–Urban Disparities in HPV Vaccination Coverage Among Adolescents in the Central Part of the State of Illinois, USA. J Community Health. 2022;48(1):24–29. doi:10.1007/s10900-022-01136-x [52]	2022	Midwest	2015–2020	Quantify the rates of HPV vaccine initiation and completion in an academic medical center in central Illinois and identify factors associated with both outcomes	Retrospective Chart Review	9351	Adolescents		HPV vaccine initiation and completion		Vaccine initiation for HPV was 46.2% and completion was 24.7% among the participants. Older age and being female increased the odds of initiating and completing the HPV vaccination. Adolescents residing in rural areas were 38% and 24% less likely to initiate (aOR = 0.62; 95 CI: 0.54–0.72) and complete (aOR = 0.76, 95 CI: 0.65–0.88) the HPV vaccine compared to those in urban areas. Adolescents were less likely to initiate and complete the HPV vaccine if they were not up-to-date on the hepatitis A, meningococcal, and TDaP vaccines.	
Adjei Boakye E, McKinney SL, Whittington KD, et al. Association between Sexual Activity and Human Papillomavirus (HPV) Vaccine Initiation and Completion among College Students. Vaccines. 2022;10(12):2079. doi:10.3390/vaccines10122079 [53]	2022	Midwest	2021	Examine if sexual activity was associated with HPV vaccination uptake among university students	Cross-sectional study	951	Adults		HPV vaccine initiation and completion		Students who had ever engaged in sexual activity were more likely to have initiated (aOR = 2.06, 95% CI: 1.34–3.17) the HPV vaccine; however, no difference was observed for HPV vaccine completion.	
Askelson N, Ryan G, McRee AL, et al. Using concept mapping to identify opportunities for HPV vaccination efforts: Perspectives from the Midwest and West Coast. Eval Program Plann. 2021;89:102010. doi:10.1016/j.evalprogplan.2021.102010 [54]	2021	Midwest, West	2018–2019	Solicit perspectives on barriers and facilitators to HPV vaccination from state-level stakeholders	Observational	134	Other (stakeholders)		Barriers and facilitators to HPV vaccination from state-level stakeholders		Clusters rated most feasible included coordinated/consistent messaging and education. Clusters rated as most important for improving vaccination in rural areas were education (Mean [M] = 4.21), provider influence (M = 4.10), and evidence-based interventions (M = 4.07). All items except coordinated/consistent messaging were rated as more important than feasible.	
Askelson NM, Campo S, Lowe JB, Smith S, Dennis LK, Andsager J. Using the Theory of Planned Behavior to Predict Mothers’ Intentions to Vaccinate Their Daughters Against HPV. J Sch Nurs. 2010;26(3):194–202. doi:10.1177/1059840510366022 [55]	2010	Midwest	2007	Investigate the influences of mothers’ intentions to vaccinate their daughters against HPV	Cross-sectional survey	217	Parents of adolescents	Theory of Planned Behavior	Intention to vaccinate		Attitudes were the strongest predictor of mothers’ intentions to vaccinate (β = 0.61, *p* < 0.001). Mothers with subjective norms that were in support of the vaccine were more likely to intend to vaccinate (β = 0.16, *p* < 0.05).	
Askelson NM, Campo S, Smith S, Lowe JB, Dennis L, Andsager J. Assessing physicians’ intentions to talk about sex when they vaccinate nine-year-old to 15-year-old girls against HPV. Sex Educ. 2011;11(4):431–441. doi:10.1080/14681811.2011.595252 [56]	2011	Midwest	Not listed	Assess whether physicians would use HPV vaccination to communicate with young female patients about sex	Cross-sectional study	207	Provider	Theory of Planned Behavior	Intention to vaccinate		Most physicians intended to talk about sexually transmitted infections when they vaccinate against HPV (90.3%). Physicians’ intentions to talk about sex are influenced by attitudes (β = 0.18, *p* < 0.05), subjective norms (β = 0.53, *p* < 0.001), and perceived behavioral control (β = 0.15, *p* < 0.05).	
Askelson NM, Ryan G, Seegmiller L, Pieper F, Kintigh B, Callaghan D. Implementation Challenges and Opportunities Related to HPV Vaccination Quality Improvement in Primary Care Clinics in a Rural State. J Community Health Aug. 2019;44(4):790–795. doi:10.1007/s10900-019-00676-z [57]	2019	Midwest	2017	Understand the decision-making process of intervention selection and implementation from the perspective of Vaccine for Children (VHC) liaisons	Cross-sectional study	115	Clinics		How HPV intervention selection decisions are made and the extent of implementation		Respondents (VFC liaisons) reported decisions about vaccine QI were made by multiple actors within their own clinics (45.1%), by a clinic manager in charge of multiple clinics (33.0%) and/or at a centralized administrative office (35.2%). Additionally, the majority of respondents considered external actors, like insurance companies (52.7%) or Medicaid/Medicare (50.5%), important to the decision-making process.	
Ayres S, Gee A, Kim S. Human Papillomavirus Vaccination Knowledge, Barriers, and Recommendations Among Healthcare Provider Groups in the Western United States. J Cancer Educ Dec. 2022;37(6):1816–1823. doi:10.1007/s13187-021-02047-6 [58]	2021	West	2019	Compare differences in same-day HPV vaccination recommendation at clinics Mountain West (MW) in states between healthcare provider and staff groups, and compare different provider groups’ perceived challenges associated with HPV vaccination, HPV vaccination knowledge, HPV recommendation practices, and same-day HPV vaccination recommendation	Cross-sectional study	99	Providers		Provider challenges and knowledge, and recommendation practices, and same-day HPV vaccination		Clinicians had a higher knowledge of HPV vaccination, identified more challenges that limit HPV vaccination, and had better HPV recommendation practices. There was no difference between clinicians and OTMs on the tendency of the patients to receive vaccine on same day as recommended. No significant differences were found between rural and urban subgroups on demographics or survey responses.	
Beck A, Bianchi A, Showalter D. Evidence-Based Practice Model to Increase Human Papillomavirus Vaccine Uptake: A Stepwise Approach. Nurs Womens Health. 2021;25(6):430–436. doi:10.1016/j.nwh.2021.09.006 [59]	2021	South	2018–2019	Increase uptake of HPV vaccination by implementing HPV education along with a strong provider recommendation to parents of youth and adolescents	Controlled Trial	24	Clinic	Evidence-based practice model	HPV vaccine initiation and completion		Of all the 24 vaccine-eligible patients, all 24 ended up receiving initiation of the vaccine or completed a previously started series.	
Bednarczyk RA, Whitehead JL, Stephenson R. Moving beyond sex: Assessing the impact of gender identity on human papillomavirus vaccine recommendations and uptake among a national sample of rural-residing LGBT young adults. Papillomavirus Res. 2017;3:121–125. doi:10.1016/j.pvr.2017.04.002 [60]	2017	National	2014	Compare HPV vaccine recommendation and uptake by self-reported sex assigned at birth and current gender identity	Cross-sectional survey	660	Adults		Healthcare provider HPV vaccine recommendation and HPV vaccine Initiation		Receipt of HPV vaccination recommendation and at least one HPV vaccine dose was higher for female SAAB (47% and 44%, respectively) compared to male SAAB (17% and 14%, respectively), as well as female or transmale gender identity compared to male or transfemale gender identity. Approximately half of vaccinated respondents reported receiving HPV vaccine between 13 and 17 years of age.	
Berenson AB, Hirth JM, Kuo YF, Rupp RE. Quantitative and qualitative assessment of an all-inclusive postpartum human papillomavirus vaccination program. Am J Obstet Gynecol. 2021;224(5):504.e1–504.e9. doi:10.1016/j.ajog.2020.11.033 [61]	2021	South	2012–2019	Examine the success and limitations of a program that promotes HPV vaccination to young adult women postpartum after expansion	Mixed methods	6961	Other (young postpartum women)		HPV vaccine completion		In the initial program, 76.9% completed the series, and in the expansion program, 73.5% completed the series.	
Blake KD, Ottenbacher AJ, Finney Rutten LJ, et al. Predictors of Human Papillomavirus Awareness and Knowledge in 2013. Am J Prev Med. 2015;48(4):402–410. doi:10.1016/j.amepre.2014.10.024 [62]	2015	National	2013	Assess current population awareness of and knowledge about HPV and the HPV vaccine and the contribution of sociodemographic characteristics to disparities in HPV awareness and knowledge.	Cross-sectional Survey	3103	Adults		HPV and HPV vaccine awareness and knowledge	Sociodemographic characteristics associated with HPV knowledge/awareness	68% had heard of HPV and the HPV vaccine, and 62% knew that HPV causes cervical cancer.	Age and sex impacted awareness and knowledge of HPV and the vaccine. Education, race, health insurance access, and internet access affected HPV and vaccine awareness, while rurality, education, and race affected some HPV knowledge questions. Those in rural areas were less likely than those in urban areas to know that HPV causes cervical cancer [aOR = 0.54 (0.30–0.98), *p* < 0.05].
Boitano TKL, Daniel C, Kim Y il, Straughn JM, Peral S, Scarinci I. Beyond words: Parental perceptions on human papillomavirus vaccination recommendations and its impact on uptake. Prev Med Rep. 2021;24:101596. doi:10.1016/j.pmedr.2021.101596 [63]	2021	South	2019–2020	Evaluate the impact of provider recommendations regarding HPV vaccination uptake in a rural setting	Cross-sectional survey	368	Parents		HPV vaccine Initiation and Intention to vaccinate		Approximately 40% indicated receiving a recommendation from a provider to vaccinate their child. Parental impression from the recommendation of HPV vaccination being “important” was significantly associated with the child being vaccinated that day (OR = 7.31, 95% CI: 2.20–24.3) as well as scheduling HPV vaccination (OR = 3.17, 95% CI: 1.01–9.92). Parents who got the impression that “there was no hurry” were less likely to vaccinate their child that day (OR = 0.23, 95% CI: 0.09–0.59).	
Boyce TG, Christianson B, Hanson KE, et al. Factors associated with human papillomavirus and meningococcal vaccination among adolescents living in rural and urban areas. Vaccine X. 2022;11:100180. doi:10.1016/j.jvacx.2022.100180 [64]	2022	Midwest	2019	Assess factors and barriers associated with adolescent HPV and MenACWY vaccination to understand the determinants of rural–urban differences	Cross-sectional study	536	Parents		Parents' perception of importance placed on HPV vaccine by HCP and HPV vaccine initiation		60% of teens received one or more doses of HPV vaccine. Among teens who received Tdap, HPV, and MenACWY, more rural teens received the three vaccines on the same day than urban teens (62% vs. 44%, *p* = 0.02). Fewer rural parents reported discussion with their provider and HPV vaccination as being “very important” for their teen according to their provider (45% vs. 54%, *p* = 0.08). The HPV vaccine harms factor had the lowest mean score (least favorable toward vaccination) among the factors assessed and differed by residency. Mean HPV vaccine harms score was significantly lower among rural parents than urban parents (5.49 (SD = 2.32) vs. 6.05 (SD = 2.35), *p* = 0.006).	
Brennan LP, Rodriguez NM, Head KJ, Zimet GD, Kasting ML. Obstetrician/gynecologists’ HPV vaccination recommendations among women and girls 26 and younger. Prev Med Rep. 2022;27:101772. doi:10.1016/j.pmedr.2022.101772 [65]	2022	National	2019	Identify the factors that are most associated with an OB/GYN being a strong and frequent HPV vaccine recommender to girls and women 26 years of age or younger	Cross-sectional study	205	Providers	Competing Demands Model	Strength and frequency of provider recommendations		56.3% (n = 116) were categorized as strong and frequent recommenders of the HPV vaccine. The clinic-level attributes were having the vaccine stocked (aOR = 2.66, 95%CI:1.02–6.93) and suburban (aOR = 3.31, 95%CI:1.07–10.19) or urban (aOR = 3.54, 95%CI:1.07–11.76) location versus rural for strong and frequent vaccine recommendations. Being a strong and frequent recommender was positively associated with believing other gynecologists frequently recommend the vaccine (aOR = 24.33, 95%CI:2.56–231.14) and believing that 50% or more of their patients are interested in receiving the vaccine (aOR = 2.77, 95%CI: 1.25–6.13).	
Brewer NT, Hall ME, Malo TL, Gilkey MB, Quinn B, Lathren C. Announcements Versus Conversations to Improve HPV Vaccination Coverage: A Randomized Trial. Pediatrics. 2017;139(1). doi:10.1542/peds.2016-1764 [66]	2017	South	2015	Determine the effectiveness of training providers to improve their recommendations using either presumptive “announcements” or participatory “conversations”	randomized clinical trial	17,173	Providers		HPV vaccine initiation	Six-month difference in HPV vaccination coverage for 13–17-year-olds and 3-month difference in all measures	5.4% (95% CI: 1.1–9.7%) increase in vaccination initiation for patients who received announcement training compared to control clinics for 11–12-year-olds after 6 months. Conversation training did not differ from control clinics	At 6 months, neither announcement or conversation training was effective for changing coverage for other vaccination outcomes or for adolescents aged 13 through 17. After 3 months, clinics' announcement training had higher HPV initiation rates compared to control clinics.
Britt R, Britt BC. The need to develop health campaigns for obtaining the HPV vaccine in rural and medically underserved college campuses. Educ Health. 2016;34:74–78 [67]	2016	Midwest	Not listed	Examine behavioral factors and their association with HPV vaccination	Cross-sectional study	327	Adults	Theory of Planned Behavior	Intention to vaccinate		There was no significant relationship between gender, intent, normative beliefs, or attitudes towards receiving the HPV vaccine. Neither attitudes nor perceived behavioral control were identified as significant predictors of intent, but subjective norms did serve as a significant predictor of receiving the HPV vaccine.	
Britt RK, Englebert AM. Behavioral determinants for vaccine acceptability among rurally located college students. Health Psychol Behav Med. 2018;6(1):262–276. doi:10.1080/21642850.2018.1505519 [68]	2018	South	Not listed	Investigate the demands of family, school, social, and work and the potential relationships and their potential impact on attitudes, subjective norms, and perceived behavioral control related to vaccination uptake	Cross-sectional study	208	Adults	Theory of Planned Behavior	Intention to vaccinate and HPV vaccine initiation		Attitudes towards vaccination uptake were positively related to increased work demands (r = 0.223, *p* < 0.001). Subjective norms were not significant with any variable. PBC and vaccination uptake were associated with work demands (r = 0.168, *p* < 0.001), school demands (r = 0.227, *p* < 0.01), and social demands (r = 0.056, *p* < 0.001). Intent to vaccinate was predicted by work demands (r = 0.143, *p* < 0.01), school demands (r = 0.130, *p* < 0.01), and social demands (r = 0.080, *p* < 0.01).	
Brumbaugh JT, Sokoto KC, Wright CD, et al. Vaccination intention and uptake within the Black community in Appalachia. Health Psychol. 2023;42(8):557–566. doi:10.1037/hea000126 [69]	2023	South	2020	Identify and compare psychosocial predictors of COVID-19, flu, and HPV vaccination intention or behavior	Cross-sectional study	336	Adults	Andersen model	HPV vaccine initiation		Age was negatively associated (OR = 0.96, *p* = 0.023) and vaccine confidence was positively associated with HPV vaccination uptake (OR = 1.77, *p* < 0.001). Vaccine calculation remained significantly associated with HPV vaccination uptake in the final step of the overall model (OR = 1.32, *p* = 0.050)	
Carman AL, McGladrey ML, Goodman Hoover A, Crosby RA. Organizational Variation in Implementation of an Evidence-Based Human Papillomavirus Intervention. Am J Prev Med. 2015;49(2):301–308. doi:10.1016/j.amepre.2015.03.011 [70]	2015	South	2013–2014	Implement the 1-2-3 Pap intervention in a public health setting and identify site-specific variations in its implementation	Quasi-experimental (pre- and post-implementation study)	18	Other (health departments)		HPV vaccine initiation and Implementation outcome: organizational readiness for change		The ORCA revealed variation in implementation strategies was widespread despite the “controls” provided by each site receiving the same instructions, incentives, and technical assistance. There was no statistical difference between ORCA scores and either channel selection or vaccine uptake. Among female patients, clinics using the waiting room channel had a mean total dose of 17.40. For clinics using the Internet distribution channel, the mean was 36.92. Interviews reinforced that there were wide implementation strategies among the LHD	
Cataldi JR, Brewer SE, Perreira C, et al. Rural Adolescent Immunization: Delivery Practices and Barriers to Uptake. J Am Board Fam Med. 2021;34(5):937–949. doi:10.3122/jabfm.2021.05.210107 [71]	2021	West	2019	Assess whether there were rural–urban differences in perceived parental vaccine confidence and beliefs, and adolescent immunization delivery practices among Colorado vaccine providers	Cross-sectional study	437	Providers	Health belief model	Barriers to adolescent vaccination and perceived parental vaccine attitudes and immunization practices	Percentage of clinicians that think parents would agree with vaccine benefits	Rural respondents were less likely than urban respondents to agree that most patients have insurance that covers vaccination (86% vs. 97%; *p* = 0.02). Rural respondents were less likely than urban respondents to indicate most parents in their practice would agree with statements about vaccine benefits (*p* = 0.02) and trust in medical providers (*p* = 0.05). Fewer providers strongly recommended HPV vaccine (81% for females, 80% for males 11 to 12 years) than other adolescent immunizations (Tdap, MenACWY, influenza: 87–97%). There were no significant differences between rural and urban responses for perceived parental HPV vaccination beliefs.	
Cates JR, Ortiz RR, North S, Martin A, Smith R, Coyne-Beasley T. Partnering with middle school students to design text messages about HPV vaccination. Health Promot Pract. 2015 Mar;16(2):244–55. doi: 10.1177/1524839914551365. Epub 2014 Sep 25. PMID: 25258431; PMCID: PMC5319196. [72]	2015	South	2011–2012	Examine the acceptability of text messages about HPV vaccination and message preferences among adolescents	Cross-sectional survey	43	Adolescents	Health Belief Model	Preferences for proposed text messages about HPV and HPV vaccine	Acceptability of using text messages to convey HPV vaccine information	More than 70% used text messaging with a cell phone. The text message with the best composite score (M = 2.33, SD = 0.72) for likeability, trustworthiness, and motivation to seek more information was a gain frame emphasizing reduction in HPV infection if vaccinated against HPV. Text messages with lower scores emphasized threats of disease if not vaccinated.	Participants (68%) preferred doctors as their information source.
Cates JR, Shafer A, Diehl SJ, Deal AM. Evaluating a County-Sponsored Social Marketing Campaign to Increase Mothers’ Initiation of HPV Vaccine for Their Preteen Daughters in a Primarily Rural Area. Soc Mark Q. 2011;17(1):4–26. doi:10.1080/15245004.2010.546943 [73]	2012	South	2009	Evaluate a social marketing campaign initiated by 13 North Carolina counties to raise awareness among parents and reduce barriers to accessing the vaccine in a primarily rural area	Quasi-experimental	294	Parents of adolescents and healthcare providers	Health Belief Model	HPV vaccine initiation	Awareness of Media campaign	HPV vaccination rates within six months of campaign launch were 2% higher for 9–13-year-old girls in two of the four intervention counties compared to 96 non-intervention counties.	Most respondents (82%) were aware of HPV messages, logos, or both. Overall awareness did not differ by daughters’ age, mother’s race, income level, or rural/urban residence. Mothers in the target age group were less likely to see posters “frequently” or “occasionally” than mothers with older daughters (44% vs. 69%, *p* < 0.05). Of respondent providers (n = 35), 94% used campaign brochures regularly or occasionally in conversations with parents.
Chido-Amajuoyi OG, Jackson I, Yu R, Shete S. Declining awareness of HPV and HPV vaccine within the general US population. Hum Vaccines Immunother. 2020;17(2):420–427. doi:10.1080/21645515.2020.1783952 [74]	2020	National	2008–2018	Determine awareness of HPV and HPV vaccine in the US over the 10-year period	Cross-sectional survey	21,325	Adults		HPV and HPV vaccine awareness	Sociodemographic factors affecting awareness over time	The awareness of HPV decreased by 4.4%, and HPV vaccine awareness declined by 4.9% over time.	The lowest awareness was among racial minorities, rural residents, male respondents, those aged 65 years and older, as well as those with the lowest educational and socioeconomic standing
Cunningham-Erves J, Koyama T, Huang Y, et al. Providers’ Perceptions of Parental Human Papillomavirus Vaccine Hesitancy: Cross-Sectional Study. JMIR Cancer. 2019;5(2):e13832. doi:10.2196/13832 [75]	2019	South	2018	Characterize the reasons for and level of parental HPV vaccine hesitancy as perceived by pediatric providers in Middle Tennessee and identify provider-level and clinic-level factors influencing perceived parental hesitancy	Cross-sectional survey	187	Providers		Perceived parental barriers to HPV vaccine hesitancy among pediatric providers		The most common parental barriers to HPV vaccination Perceived by providers were concerns about HPV vaccine safety (88%), child being too young (78%), low risk of HPV infection for child through sexual activity (70%), and mistrust in vaccines (59%). Perceived parental HPV vaccine hesitancy was significantly associated with several provider-level factors: self-efficacy (*p* = 0.001), outcome expectations (*p* < 0.001), and confidence in HPV vaccine safety (*p* = 0.009).	
Dang JHT, McClure S, Gori ACT. Implementation and evaluation of a multilevel intervention to increase uptake of the human papillomavirus vaccine among rural adolescents. J Rural Health Jan. 2023;39(1):136–141. doi:10.1111/jrh.12690 [76]	2023	West	2018–2020	Evaluate the effectiveness of a multilevel evidence-based intervention aimed at increasing HPV vaccination coverage among rural adolescent patients in a rural health clinic	Controlled Trial	498	Adolescents		Initiation and completion		Adolescent patients ages 11–17 who had initiated the HPV vaccine series (82.7% vs. 52.4%, *p* < 0.0001) and completed the vaccine series (58.0% vs. 27.0%, *p* < 0.0001) were significantly greater at follow-up compared to baseline.	
Daniel CL, Lawson F, Vickers M, et al. Enrolling a rural community pharmacy as a Vaccines for Children provider to increase HPV vaccination: a feasibility study. BMC Public Health. 2021;21(1). doi:10.1186/s12889-021-11304-8 [77]	2021	South	2019–2020	Examine the feasibility and potential effectiveness of enrolling a rural, community pharmacy as a VFC provider	pilot study	1	HPV-eligible community members		HPV vaccine initiation	Pharmacy VFC enrollment feasibility measures	166 vaccines were administered to 89 adolescents, which included 55 HPV doses, 53 Tdap doses, 45 Meningococcal doses, and 13 Influenza doses. 64% (64) were VFC patients. The VFC intervention had positive feedback in the community and improved access to VFC-approved providers	The pharmacy increased overall prescription revenue by 34.1% (compared to a 6.9% increase for this time period in the previous year) and had a 17.8% increase in Medicaid prescriptions filled, thought to be heavily influenced by the added Medicaid/VFC services. Total revenue increased 24.4% after introduction of the intervention, compared to an 8.0% increase the previous year
Fernandez-Pineda M, Cianelli R, Villegas N, et al. Preferred HPV and HPV Vaccine Learning Methods to Guide Future HPV Prevention Interventions Among Rural Hispanics. J Pediatr Nurs. 2021;60:139–145. doi:10.1016/j.pedn.2021.04.026 [78]	2021	South	Not listed	Determine rural Hispanic parents’ preferred HPV and HPVV learning methods	qualitative	23	Parents		Rural Hispanic parents’ preferred HPV and HPVV learning methods		For parents, small educational sessions (“charlas”) were the most preferred way to learn about HPV and HPVV. Other possible modes were healthcare providers, community-wide campaigns, mail, pharmacy, radio/tv, word of mouth, research studies, CDs/DVDs, email, pamphlets, social media videos, and webpage posts. For families/children to learn about HPV and HPVV, school-based events were most preferred. Other modes included healthcare providers/teachers, healthcare centers/clinic short video clips, health fairs, educational sessions, telephone(texts), and social media posts.	
Fish LJ, Harrison SE, McDonald JA, et al. Key stakeholder perspectives on challenges and opportunities for rural HPV vaccination in North and South Carolina. Hum Vaccines Immunother. 2022;18(5). doi:10.1080/21645515.2022.2058264 [79]	2022	South	2019–2020	Learn about barriers and opportunities to scaling up adolescent vaccination, including HPV vaccination, in rural areas	qualitative	14	Other (stakeholders)	Social Ecological framework	Key stakeholder perspectives on challenges to HPV vaccination in rural areas		Individual: misinformation/vaccine beliefs and attitudes to preventive care; Provider: provider shortage, hard to participate in VFC programs, lack of strong provider HPV vaccine recommendations; System: no state mandate for HPV vaccine and school enrollment, school nurses could help address provider gaps, expand current programs for adolescents to include vaccines	
Ford M, Cartmell K, Malek A, et al. Evaluation of the First-Year Data from an HPV Vaccination Van Program in South Carolina, U.S. J Clin Med. 2023;12(4):1362. doi:10.3390/jcm12041362 [80]	2023	South	2021–2022	Assess the program’s effectiveness by increasing the HPV vaccine uptake in SC	observational	552	Adolescents and Adults		HPV vaccine initiation		552 participants received vaccinations from the HPV Van Program with 243 of them receiving the HPV vaccine	
Gilbert PA, Lee AA, Pass L, et al. Queer in the Heartland: Cancer Risks, Screenings, and Diagnoses among Sexual and Gender Minorities in Iowa. J Homosex. Published online October 19, 2020:1–17. doi:10.1080/00918369.2020.1826832 [81]	2020	Midwest	2017	Develop detailed epidemiologic profiles of Iowa’s SGM for cancer prevention	Cross-sectional study	567	Adults		HPV vaccine initiation		Less than half (41.8%) of those plausibly eligible individuals reported HPV vaccine initiation. The majority (80.0%) reported receiving two or three doses. Compared to ciswomen, cismen had 78% lower odds of reporting HPV vaccination initiation (OR = 0.22, 95% CI: 0.11–0.45) but there was no difference for transgender/genderqueer individuals. Examining sexual orientation differences, bisexual/pansexual respondents had over four-times higher odds and queer/other individuals had 2 times higher odds of reporting HPV vaccination initiation compared to gay/lesbian respondents (OR = 4.34, 95% CI: 2.18–8.62 and OR = 2.10, 95% CI: 1.11–3.97, respectively).	
Goessl CL, Christianson B, Hanson KE, et al. Human papillomavirus vaccine beliefs and practice characteristics in rural and urban adolescent care providers. BMC Public Health. 2022;22(1). doi:10.1186/s12889-022-13751-3 [82]	2022	Midwest	2019	Identify the HPV vaccine attitudes and practices that were most strongly associated with rural vs. urban providers	Cross-sectional survey	437	Providers		Provider HPV Vaccine Resources, Practices and Attitudes		Five vaccine factors were different between rural and urban providers, including evening/weekend appointments (aOR = 0.21, 95% CI: 0.12, 0.36), standing vaccination orders (aOR = 2.81, 95% CI: 1.61, 4.91), prior experience with vaccine quality improvement projects (aOR = 0.52, 95% CI: 0.28, 0.98), providing HPV vaccine information before it is due (aOR = 3.10, 95% CI: 1.68, 5.71), and recommending HPV vaccine during urgent care visit (aOR = 0.37, 95% CI: 0.18, 0.79). Other practices and attitudinal exposures were statistically similar between rural and urban providers.	
Gunn R, Ferrara LK, Dickinson C, et al. Human Papillomavirus Immunization in Rural Primary Care. Am J Prev Med. 2020;59(3):377–385. doi:10.1016/j.amepre.2020.03.018 [83]	2020	West	2018	Identify the organizational structures and clinical workflows that enable rural, high-performing primary care clinics to support HPV vaccine delivery	mixed methods	12	Clinics	Positive Deviance framework	organizational structures and workflows		Four key themes were identified: (1) standardized workflows to identify patients due for the vaccine and had vaccine administration protocols, (2) have a vaccine champion, (3) clinical staff were comfortable providing immunizations regardless of visit type, and (4) clear, persuasive language to recommend or educate parents/patients about the vaccine’s importance	
Harris KL, Tay D, Kaiser D. The perspectives, barriers, and willingness of Utah dentists to engage in human papillomavirus (HPV) vaccine practices. Hum Vaccin Immunother. 2020;16(2):436–444. doi:10.1080/21645515.2019.1649550 [84]	2019	West	2017–2018	Examine the relationship between dental providers’ perspectives about their scope of practice, barriers, and willingness to engage and collaborate in HPV vaccination practices in the dental setting	Cross-sectional Survey	203	Other (Dentists)		Barriers to HPV vaccine among dentists and dentists’ willingness to engage in HPV vaccination practices and collaborate with primary care providers.		Majority of Utah dentists surveyed perceived that discussing the link between HPV and OPC and recommending the HPV vaccine is within their scope of practice, but not administration of the HPV vaccine. A significantly higher proportion of urban Utah dentists disagreed that they were concerned about the safety of the HPV vaccine (n = 141, 73.43%, *p* = 0.011), or that they were concerned about the liability related to the HPV vaccine (n = 103, 53.34%, *p* = 0.004) were compared with rural dental providers (n = 13, 6.77%; n = 13, 6.77%). Discussing, recommending, and administering the HPV vaccine did not significantly differ by dentists’ age group, rurality, time spent on patient education, or length of dental experience.	
Harry ML, Asche SE, Freitag LA, et al. Human Papillomavirus vaccination clinical decision support for young adults in an upper midwestern healthcare system: a clinic cluster-randomized control trial. Hum Vaccines Immunother. 2022;18(1). doi:10.1080/21645515.2022.2040933 [85]	2022	Midwest	2018–2019	Test Clinical Decision Support with or without shared decision-making tools (SDMTs) on HPV vaccination rates compared to usual care (UC)	Randomized controlled trial	34 clinics	Adults		HPV vaccine completion		The HPV vaccination series was completed by 12 months in 2.3% (95% CI: 1.6–3.2%) of CDS, 1.6% (95% CI: 1.1–2.3%) of CDS + SDMT, and 2.2% (95% CI: 1.6–3.0%) of UC patients, and at least one HPV vaccine was received by 12 months in 13.1% (95% CI: 10.6–16.1%) of CDS, 9.2% (95% CI: 7.3–11.6%) of CDS + SDMT, and 11.2% (95% CI: 9.1–13.7%) of UC patients.	
Hatch BA, Valenzuela S, Darden PM. Clinic-level differences in human papillomavirus vaccination rates among rural and urban Oregon primary care clinics. J Rural Health Mar. 2023;39(2):499–507. doi:10.1111/jrh.12724 [86]	2023	West	2019	Compare HPV vaccination between rural and urban primary care clinics and examine the association of rurality with HPV vaccination	Cross-sectional study	537	Clinics		HPV vaccine initiation and completion		The mean rate of HPV vaccine ≥ 1 dose was lower among rural clinics (46.9% vs. 51.1%, *p* = 0.039), as was vaccination UTD (40.5% vs. 49.9%, *p* < 0.001) when compared to urban clinics. The rural/urban disparity was not significant after adjusting for other individual- and clinic-level characteristics.	
Henry KA, Swiecki-Sikora AL, Stroup AM, Warner EL, Kepka D. Area-based socioeconomic factors and Human Papillomavirus (HPV) vaccination among teen boys in the United States. BMC Public Health. 2017;18(1). doi:10.1186/s12889-017-4567-2 [87]	2017	National	2012–2013	Examine associations between both individual-level and area-based factors and HPV vaccine initiation and completion among boys	Secondary data analysis	19,518	Adolescents		HPV vaccine initiation and completion		Area-based poverty was not statistically significantly associated with HPV vaccination initiation, but it was associated with completion, with boys living in high-poverty areas having higher odds of completing the series than boys in low-poverty areas. Boys from urban or densely populated areas have higher odds of initiation and completion compared to boys living in non-urban, less densely populated areas.	
Jafari SDG, Appel SJ, Shorter DG. Risk Reduction Interventions for Human Papillomavirus in Rural Maryland. J Dr Nurs Pract. 2020;13(2):134–141. doi:10.1891/jdnp-d-19-00047 [88]	2020	South	2017–2018	Address patient or parental perceptions Leading to vaccine hesitancy and identify the vaccine impact from provider to patient education	Mixed methods	416	Adolescents/ providers		HPV vaccine initiation		A documentary movie for women aged 12–26 was implemented to decrease HPV-related risks; the impact was not significant. Direct provider to patient recommendations resulted in a 15% increase in HPV immunizations.	
Kepka D, Christini K, McGough E, et al. Successful Multi-Level HPV Vaccination Intervention at a Rural Healthcare Center in the Era of COVID-19. Front Digit Health. 2021;3. doi:10.3389/fdgth.2021.719138 [89]	2021	West	2019–2021	Test HPV vaccination intervention that includes healthcare team training activities and patient reminders to reduce missed opportunities and improve the rate of appointment scheduling for HPV vaccination in a rural medical clinic	Quasi-experimental study	402	Parents, adolescents and adults		Missed opportunities for HPV vaccination		Missed opportunities for HPV vaccination declined significantly between the pre-intervention and the post-intervention period (21.6 vs. 8.1%, respectively, *p* = 0.002). Participants who recalled receipt of a vaccination reminder had 7.0 (95% CI 2.4–22.8) times higher unadjusted odds of scheduling a visit compared with those who did not recall receiving a reminder. The unadjusted odds of confirming that they had scheduled or were intending to schedule a follow-up appointment to receive the HPV vaccine were 4.9 (95% CI 1.51–20.59) times greater among those who had not received the vaccine for themselves or for their child.	
Kepka D, Coronado GD, Rodriguez HP, Thompson B. Evaluation of a Radionovela to Promote HPV Vaccine Awareness and Knowledge Among Hispanic Parents. J Community Health. 2011;36(6):957–965. doi:10.1007/s10900-011-9395-1 [90]	2011	West	2008–2009	Investigate the efficacy of messages delivered via a radionovela to improve HPV and HPV vaccine-related knowledge and attitudes	randomized controlled trial	88	Parents		HPV and HPV vaccine awareness and knowledge & attitudes/beliefs		Parents who listened to the HPV radionovela (intervention group) were more likely to confirm that HPV is a common infection (70% vs. 48%, *p* = 0.002), to deny that women are able to detect HPV (53% vs. 31%, *p* = 0.003), to know vaccine age recommendations (87% vs. 68%, *p* = 0.003), and to confirm multiple doses (48% vs. 26%, *p* = 0.03) than control group parents.	
Kepka DL, Ulrich AK, Coronado GD. Low Knowledge of the Three-Dose HPV Vaccine Series among Mothers of Rural Hispanic Adolescents. J Health Care Poor Underserved. 2012;23(2):626–635. doi:10.1353/hpu.2012.0040 [91]	2012	West	2009	Investigate correlates of HPV vaccine uptake by adolescent daughters of rural Hispanic mothers	Cross-sectional survey	78	Parents	Social Ecological Framework	HPV vaccine initiation		Mothers who had heard of the HPV vaccine were more likely to have a vaccinated daughter (*p* < 0.01). Mothers who thought their daughter’s father would approve were more likely to have a vaccinated daughter (*p* = 0.004). Parents who believed that only one injection was necessary were more likely to have a vaccinated daughter (*p* = 0.009)	
Kim S, Zhou K, Parker S, Kline KN, Montealegre JR, McGee LU. Perceived Barriers and Use of Evidence-Based Practices for Adolescent HPV Vaccination among East Texas Providers. Vaccines. 2023;11(4):728. doi:10.3390/vaccines11040728 [92]	2023	South	2022	Understand current clinical practices regarding HPV vaccination in rural East Texas primary health-care settings and assess health-care providers’ perceived barriers to HPV vaccination	Cross-sectional study	27	Clinics		Perceived barriers to HPV vaccination in clinics and strategies used by clinics to increase HPV vaccination rates	HPV vaccine-promoting clinical practices	The most prevalent perceived barrier was missed opportunities for vaccination (66.7%), and concern about vaccine hesitancy (44.4%) because of the pandemic.	Many clinics surveyed currently implement evidence-based practices to promote HPV vaccination, but using a “refusal to vaccinate” form (29.6%), having an identified HPV vaccine champion (29.6%), and recommending the HPV vaccine at age 9 (22.2%) were least implemented among these clinics.
Koskan AM, Dominick LN, Helitzer DL. Rural Caregivers’ Willingness for Community Pharmacists to Administer the HPV Vaccine to Their Age-Eligible Children. J Cancer Educ Feb. 2021;36(1):189–198. doi:10.1007/s13187-019-01617-z [93]	2021	south	Not listed	Explore rural caregivers’ perceptions of receiving the HPV vaccine from their local pharmacist and determine preferences for education for both the vaccine and receiving vaccines from pharmacists	Qualitative	26	Providers		Caregivers’ perceptions of the HPV vaccine and their willingness for pharmacist- administered HPV vaccination	Awareness about the HPV vaccine, HPV vaccine barriers, and facilitators.	Most caregivers were unaware that pharmacists could offer adolescent vaccines, but most were willing to allow their children to receive the vaccine from this non-traditional source. The primary concern was pharmacist training for administering the HPV vaccine.	Caregivers preferred print fliers disseminated in various locations and Facebook for channels of health education about HPV vaccine availability in pharmacies.
Kurani S, MacLaughlin KL, Jacobson RM, et al. Socioeconomic disadvantage and human papillomavirus (HPV) vaccination uptake. Vaccine. 2022;40(3):471–476. doi:10.1016/j.vaccine.2021.12.003 [94]	2022	Midwest	2016–2018	Examine HPV vaccine-related disparities by area deprivation using patient-level data from persons residing in a largely rural, Upper Midwest region	Retrospective cohort study	54,573	Adolescents		HPV vaccine initiation and completion		Individuals living in more deprived block groups were significantly less likely to initiate and complete HPV vaccinations compared to those living in the least deprived blocks. Individuals with rural residence had decreased probabilities of initiation compared to individuals living in urban areas.	
Lee HY, Luo Y, Won CR, Daniel C, Coyne-Beasley T. HPV and HPV Vaccine Awareness Among African Americans in the Black Belt Region of Alabama. J Racial Ethn Health Disparities. 2023;11(2):808–814. doi:10.1007/s40615-023-01562-0 [95]	2023	South	Not listed	Examine HPV and HPV vaccine awareness and associated factors among rural, Southern African Americans	cross-sectional survey	257	Adults		HPV and HPV vaccine awareness		Slightly more than half of the participants were aware of HPV (62.5%) and HPV vaccine (62.1%). Being single, having a family cancer history, and good self-reported health status were positively associated with both HPV and HPV vaccine awareness. Employment was positively associated with HPV awareness, and participation in social groups was positively associated with HPV vaccine awareness.	
Manganello JA, Chiang SC, Cowlin H, Kearney MD, Massey PM. HPV and COVID-19 vaccines: Social media use, confidence, and intentions among parents living in different community types in the United States. J Behav Med Apr. 2023;46(1–2):212–228. doi:10.1007/s10865-022-00316-3 [96]	2022	National	2021	Assess information seeking around children’s health and vaccines, and vaccine confidence and intention/uptake among parents living in different community types for HPV and COVID-19	Cross-sectional study	452	Parents		Intention to vaccinate	Social media use	For both HPV and COVID-19 vaccines, political affiliation was the only common factor associated with both vaccine confidence and intention/uptake. Parents who identified as Democrats compared to Republicans had greater confidence in the vaccines and had higher odds of vaccine intention/uptake for their children.	Use of Facebook was not associated with vaccine confidence.
McMann N, Trout KE. Assessing the Knowledge, Attitudes, and Practices Regarding Sexually Transmitted Infections Among College Students in a Rural Midwest Setting. J Community Health Feb. 2021;46(1):117–126. doi:10.1007/s10900-020-00855-3 [97]	2020	Midwest	2019	Assess the knowledge, attitudes, and practices regarding sexual health among rural college students in Nebraska	Cross-sectional survey	125	Adults		Knowledge, attitudes, and practices of sexual health (including percentage with HPV vaccination).		Prevalence of HPV vaccination was 51% (n = 63) and was different among females and males (60% vs. 18%, *p* < 0.001)	
Mohammed KA, Subramaniam DS, Geneus CJ, et al. Rural–urban differences in human papillomavirus knowledge and awareness among US adults. Prev Med. 2018;109:39–43. doi:10.1016/j.ypmed.2018.01.016 [98]	2018	National	2013–2017	Determine the prevalence of knowledge and awareness of HPV, the HPV vaccine, and HPV-associated cancers among rural and urban residents, and examine the association of rural/urban status with knowledge and awareness	Cross-sectional survey	10,147	Adults		Awareness, knowledge of HPV, the HPV vaccine, and HPV-associated cancers	Knowledge about HPV causing cervical, oral, anal, and penile cancers, as well as the knowledge about HPV being transmitted through sexual contact.	In comparison to rural respondents, the prevalence of awareness of HPV (67.2%; 95% CI: 67.0–69.2) and the HPV vaccine (65.8%; 95% CI: 64.2–67.1) was higher among urban respondents. Compared to urban residents, rural residents were less likely to be aware of HPV (OR = 0.68, 95% CI = 0.53–0.86) and HPV vaccine (OR = 0.78, 95% CI = 0.63–0.97).	Additionally, the prevalence of knowing that HPV causes cervical (75.4%; 95% CI: 72.5–77.3) and oral cancer (30.9%; 95% CI: 28.4–32.1), and knowing HPV is transmitted through sexual contact (65.9%; 95% CI: 63.6–67.2) was higher among urban residents than rural residents.
Morales-Campos DY, McDaniel MD, Amaro G, Flores BE, Parra-Medina D. Factors Associated with HPV Vaccine Adherence among Latino/a Adolescents in a Rural, Texas-Mexico Border County. Ethn Dis. 2022;32(4):275–284. doi:10.18865/ed.32.4.275 [99]	2022	South	2015–2018	Examine HPV vaccine initiation and completion among Hispanic adolescents in a rural, Texas-Mexico border county	Cross-sectional survey	1832	Parents	Ecological systems theory	HPV vaccine initiation and completion		Factors associated with HPV vaccine initiation and completion were female gender (*p* < 0.01), adolescent insurance status (*p* < 0.001), and receipt of required vaccines (*p* < 0.001). Adolescents who received mandatory vaccinations for school entry were five times more likely to initiate and complete the HPV vaccine series (OR = 5.39, *p* < 0.001)	
Moss JL, Gilkey MB, Reiter PL, Brewer NT. Trends in HPV Vaccine Initiation among Adolescent Females in North Carolina, 2008–2010. Cancer Epidemiol Biomarkers Prev. 2012;21(11):1913–1922. doi:10.1158/1055-9965.epi-12-0509 [100]	2012	South	2008–2010	Assess trends and disparities in HPV vaccine initiation among female adolescents in North Carolina over 3 years	Secondary Data Analysis	1427	Parents		HPV vaccine initiation		HPV vaccine initiation increased over time (2008, 34%; 2009, 41%; 2010, 44%). This upward trend was present within 11 subpopulations of girls, including those who lived in rural areas, were of minority (non-black/non-white) race, or had not recently received a preventive check-up.	
Moss JL, Gilkey MB, Rimer BK, Brewer NT. Disparities in collaborative patient–provider communication about human papillomavirus (HPV) vaccination. Hum Vaccines Immunother. 2016;12(6):1476–1483. doi:10.1080/21645515.2015.1128601 [101]	2016	National	2010	Understand how collaborative communication operates in vaccination decisions across demographic groups	Cross-sectional study	4124	Parents	Charles and Gafni framework (shared treatment decision-making model)	HPV vaccine initiation		Disparities in collaborative communication accounted for geographic variation in HPV vaccination, specifically, the higher rates of uptake in the urban/suburban vs. rural areas (*p* < 0.01). Half of parents (53%) in the survey reported collaborative communication. Poor, less educated, Spanish-speaking, Southern, and rural parents, and parents of non-privately insured and Hispanic adolescents, were least likely to report collaborative communication (all *p* < 0.05).	
Newcomer SR, Caringi J, Jones B, Coyle E, Schehl T, Daley MF. A Mixed Methods Analysis of Barriers to and Facilitators of Human Papillomavirus Vaccination Among Adolescents in Montana. Public Health Reports^®^. 2020;135(6):842–850. doi:10.1177/0033354920954512 [102]	2020	West	2013–3017	Identify barriers to and facilitators of adolescent HPV vaccination in Montana	Mixed methods	326	Adolescents	Vaccine Perceptions, Accountability and Adherence Model	HPV vaccine initiation		In Montana, initiation of the HPV vaccine series among adolescents aged 13–17 increased from 34.4% in 2013 to 65.5% in 2017. In NIS-Teen 2017 data (n = 326 adolescents), receiving a medical provider recommendation was significantly associated with series initiation (aPR = 2.3; 95% CI, 1.5–3.6). Among parents who did not intend to initiate the vaccine series for their adolescent within 12 months (n = 71), vaccine safety was the top concern (aPR = 24.5%; 95% CI, 12.1–36.9%). The two most commonly referenced themes were medical providers’ recommendation style and parental vaccine hesitancy as factors for HPV vaccination.	
Newcomer SR, Freeman RE, Albers AN, et al. Missed opportunities for human papillomavirus vaccine series initiation in a large, rural U.S. state. Hum Vaccines Immunother. 2022;18(1). doi:10.1080/21645515.2021.2016304 [103]	2022	West	2020–2021	Quantify the prevalence of missed opportunities to vaccinate adolescents against HPV when they presented for other vaccines, and to determine whether the risk of missed opportunities differed by vaccination clinics	Secondary data analysis	47,622	Adolescents		HPV vaccine initiation	Secondary: Immunization visits that were missed opportunities for initiating the human papillomavirus vaccine series for adolescents ages 11–17 years by clinic setting, Montana, 2014–2020. Tertiary: Associations between clinic setting, age, sex, and rurality with missed opportunities for initiating the human papillomavirus vaccine series for adolescents ages 11–17 years, Montana, 2014–2020	Among 47,622 adolescents, 53.9% of 71,447 vaccination visits were missed opportunities.	Receiving vaccines in public health departments was significantly associated with higher risk of missed opportunities (aRR = 1.25, 95% confidence interval = 1.22–1.27, vs. private clinics). Receipt of vaccines in Indian Health Services and Tribal clinics was associated with fewer missed opportunities (aRR = 0.72, 95% CI: 0.69–0.75, vs. private clinics).
Nguyen CG, Pogemiller MI, Cooper MT, Garbe MC, Darden PM. Characteristics of Oklahoma Pediatricians Who Dismiss Families for Refusing Vaccines. Clin Pediatr Phila Jan. 2023;62(1):24–32. doi:10.1177/00099228221108801 [104]	2023	South	2019	To assess the frequency of declining new patients or dismissing current patients who request to delay or refuse vaccines, the delay/refusal of specific vaccine(s) that prompt pediatricians to decline/dismiss patients, and the demographics of pediatricians who decline or dismiss patients	Cross-sectional study	122	Providers		Dismiss or decline for some (but not all) vaccines	Secondary: the specific vaccines causing the delay/refusal resulting in decline/dismissal. Tertiary: demographic information about physicians who decline or dismiss patients.	35% (34/98) of pediatricians dismissed current patients for refusing/delaying vaccine. 47% (48/103) declined accepting new patients due to refusing/delaying vaccine. Of the 48 physicians who declined patients, 25 (52%) declined new patients for refusing some but not all vaccines, and 23 (19%) declined new patients for refusing” all vaccines.	Secondary: Over 90% of respondents would dismiss/decline patients who refuse Dtap, Hib, PCV13, IPV, MMR, Varicella, Hep A, or Tdap. For influenza and HPV vaccines, less than 20% would dismiss or decline a patient over refusal or delay. Tertiary: More than 10 years in practice and being rural are more likely to dismiss current patients or decline new patients due to refusal for one, some, but not all, vaccines.
* Osaghae I, Darkoh C, Chido-Amajuoyi OG, et al. Healthcare Provider’s Perceived Self-Efficacy in HPV Vaccination Hesitancy Counseling and HPV Vaccination Acceptance. Vaccines. 2023;11(2):300. doi:10.3390/vaccines11020300 [105]	2022	South	2021	Examine the relationship between HPV vaccination training of HCPs and HPV vaccination status assessment and recommendation	Cross-sectional survey	1283	Providers		Provider HPV vaccination status assessment and recommendation		482 (47%) reported that they often/always assess. 537 (53%) never/sometimes assess. 756 (59%) reported they often/always recommend. 527 (41%) reported that they never/sometimes recommend.	
* Osaghae I, Darkoh C, Chido-Amajuoyi OG, et al. Association of provider HPV vaccination training with provider assessment of HPV vaccination status and recommendation of HPV vaccination. Hum Vaccines Immunother. 2022;18(6). doi:10.1080/21645515.2022.2132755 [106]	2023	South	2021	Determine the association between healthcare providers’ self-efficacy in HPV vaccination hesitancy counseling and HPV vaccination acceptance after initial and follow-up counseling sessions	Cross-sectional survey	1283	Providers		HPV vaccine initiation		HCPs who believed that they were very/completely confident in counseling HPV vaccine-hesitant parents had higher odds of observing HPV vaccination acceptance very often/always after an initial counseling session (aOR= 3.50; 95% CI: 2.25–5.44) and after follow-up counseling sessions (aOR = 2.58; 95% CI: 1.66–4.00) compared to HCPs that perceived they were not at all/somewhat/moderately confident.	
Osegueda ER, Chi X, Hall JM, Vadaparampil ST, Christy SM, Staras SAS. County-Level Factors Associated With HPV Vaccine Coverage Among 11-Year-Olds to 12-Year-Olds Living in Florida in 2019. J Adolesc Health. 2023;72(1):130–137. doi:10.1016/j.jadohealth.2022.09.005 [107]	2023	South	2019	Understand county-level characteristics associated with HPV vaccination rates	Cross-sectional study	481,846	Adolescents		HPV vaccine initiation and Completion		On average, the HPV vaccine initiation rate among the most urban counties at 65% (95%CI = 58.1–72.2) was higher than the 43% (95%CI = 36.4–50.5) HPV vaccine initiation rate among the most rural counties. HPV vaccine UTD prevalence is 21% in more urban counties and 10% for those living in rural counties.	
Panagides R, Voges N, Oliver J, Bridwell D, Mitchell E. Determining the Impact of a Community-Based Intervention on Knowledge Gained and Attitudes Towards the HPV Vaccine in Virginia. J Cancer Educ Apr. 2023;38(2):646–651. doi:10.1007/s13187-022-02169-5 [108]	2023	South	2016–2019	Compare the impact of an educational film intervention on HPV intention to vaccinate and knowledge gained in urban and rural areas; To increase knowledge and intent to receive the HPV vaccine	quasi-experimental	149	Community	Health Belief Model	Attitudes, beliefs (intention), and knowledge		Changes in knowledge about HPV were statistically significant in two out of seven questions (*p* < 0.05). Changes in attitude were statistically significant in every attitude-based question about HPV (*p* < 0.05). There were significant differences in knowledge gained and attitudes towards the HPV vaccine when comparing urban and rural locations.	
Paskett ED, Krok-Schoen JL, Pennell ML, et al. Results of a Multilevel Intervention Trial to Increase Human Papillomavirus (HPV) Vaccine Uptake among Adolescent Girls. Cancer Epidemiol Biomarkers Prev. 2016;25(4):593–602. doi:10.1158/1055-9965.epi-15-1243 [109]	2016	Midwest	2010–2015	Test the efficacy of a multilevel intervention to improve the uptake of the HPV vaccine among Appalachian girls aged 9 to 17 years old in 12 counties in Appalachian Ohio	Randomized controlled trial	456	Multilevel	Intervention guided by the Health Belief Model, Theory of Reasoned Action, Extended Parallel Process Model, and Organizational Developmental Theory	HPV vaccine initiation	HPV vaccine uptake at 6 months and uptake of the second and third HPV vaccine shots.	10 (7.7%) daughters of intervention participants received the first shot of the HPV vaccine within 3 months of being sent the intervention materials compared with 4 (3.2%) daughters of comparison group participants (*p* = 0.061). Provider knowledge about HPV increased (*p* < 0.001, from baseline to after education).	By 6 months, 17 (13.1%) daughters of intervention participants received the first HPV vaccine shot compared with eight (6.5%) daughters of comparison group participants (*p* = 0.002).
Pham D, Shukla A, Welch K, Villa A. Assessing knowledge of human papillomavirus among men who have sex with men (MSM) using targeted dating applications. Vaccine. 2022;40(36):5376–5383. doi:10.1016/j.vaccine.2022.07.048 [110]	2022	National	Not listed	Investigate knowledge regarding HPV, HPV-related cancers, and HPV vaccination rates among men who have sex with men (MSM) who had active accounts on two LGBTQ+ online dating applications	Cross-sectional survey	3342	Adults		HPV-related knowledge and HPV vaccine initiation and completion	What source has recommended the vaccine to the participant and comfort level receiving a vaccine from a dentist.	Half of the HPV vaccine-eligible respondents reported having received at least one dose of the HPV vaccine, while only 37.9% of the individuals aged 9–26 reported being vaccinated against HPV. Among the unvaccinated, 63.3% reported being interested in future vaccination, or learning more about it. There were no significant differences in vaccination status or HPV knowledge (except for cancers associated with HPV) between respondents from rural vs. urban locations.	Doctors/physicians/ nurses were reported to be the largest source of HPV vaccine recommendation. 42.2% of participants are comfortable receiving the HPV vaccine from a dentist.
Pourebrahim N, Shah P, VoPham T, et al. Time and geographic variations in human papillomavirus vaccine uptake in Washington state. Prev Med. 2021;153:106753. doi:10.1016/j.ypmed.2021.106753 [111]	2021	West	2008–2018	Identify priority areas in Washington State that have had persistently low HPV vaccine rates over time, and the contributing sociodemographic factors associated with low HPV vaccine rates	Longitudinal study	564,493	Adolescents		Initiation and completion at the census tract level		Moran’s I for HPV initiation was 0.44 and 0.47 for completion. Average vaccine initiation and completion for urban areas were higher compared to rural tracts. In urban areas, initiation rose from 11% to 34% and completion rose from 4% to 19% from 2010 to 2018. In rural areas, the rate rose from 9% to 22% initiation and 3–11% completion. Percentage of White population was positively associated with being in low vaccine areas.	
Pruitt SL, Tiro JA, Kepka D, Henry K. Missed Vaccination Opportunities Among U.S. Adolescents by Area Characteristics. Am J Prev Med. 2022;62(4):538–547. doi:10.1016/j.amepre.2021.10.014 [112]	2022	National	2015–2017	Identify adolescent-level, area-level, and household-level characteristics for coverage patterns in Tdap, HPV, and MenACWY	Secondary data analysis	63,299	Adolescents		Vaccination coverage of 1 or 2 vaccines in combination, and missed opportunity for HPV vaccine		Missed HPV vaccination opportunities were common in those in rural areas, living in the Midwest or South (not West), and not having private insurance	
Rabarison KM, Bish CL, Massoudi MS, Giles WH. Economic Evaluation Enhances Public Health Decision Making. Front Public Health. 2015;3. doi:10.3389/fpubh.2015.00164 [113]	2015	South	2010–2012	Test the cost effectiveness of 1-2-3 Pap intervention per number of completed HPV vaccine series would decrease when offered to more women in the target population	Cost Analysis	344	Adults		Implementation: cost of completion of HPV vaccine series.		Assuming the same success rate as the efficacy study, the 1-2-3 Pap adaptation scenario would cover 1000 additional women aged 18 through 26 years (344 in efficacy study; 1346 in adaptation scenario), and almost three times as many completed series (130 in efficacy study; 412 in adaptation scenario) as in the original 1-2-3 Pap efficacy study.	
Ramsay JM, Kaddas HK, Ou JY, Kepka D, Kirchhoff AC. Missed opportunities for concomitant HPV vaccination among childhood cancer survivors. Cancer Med. 2022;11(4):1181–1191. doi:10.1002/cam4.4492 [114]	2022	West	2013–2016	Assess if there are higher rates of missed opportunities to vaccinate for HPV among adolescent cancer survivors	Cohort study	2238	Adolescents		Missed opportunities for HPV vaccination		Childhood cancer survivors had more missed opportunities than the sample population (70% healthcare encounters had MOs, vs. 59%). 48.2% of the sample population and 39.8% of survivors received 1 or more doses of the HPV vaccine throughout the study. 10.2% of sample population completed the 3-dose series compared to 7.3% of the survivor group.	
Robison SG. The Impact of the Number of Injections per Visit on the Likelihood of Human Papillomavirus Immunization. J Pediatr X. 2020;3:100024. [115]	2020	West	2015–2019	Assess whether single injection visits among teens correlated with lower rates of HPV series completion, and examine whether childhood patterns of injection-limited were correlated with decreased HPV vaccine completion	Cohort study	241,453	Adolescents		Limited number of injections per visit (tdap, MenACWY, HPV), HPV initiation and completion		For adolescents who received more than 1 vaccine per visit, HPV vaccine completion rates were 62.2%, while rates were 7.7% among those considered injection-limited. 16.3% of adolescents were considered injection-limited. Of those that were not up-to-date for HPV vaccination, 76.1% were either injection-limited or did not receive their HPV vaccine at their Tdap visit. Children who had not received more than 1 injection per visit since age 4 had HPV vaccine completion rates of 3.9%.	
Rodriguez AM, Do TQN, Chen L, Schmeler KM, Montealegre JR, Kuo YF. Human papillomavirus vaccinations at recommended ages: How a middle school-based educational and vaccination program increased uptake in the Rio Grande Valley. Hum Vaccines Immunother. 2022;18(6). doi:10.1080/21645515.2022.2133315 [116]	2022	South	2016–2022	Evaluate how a community-based education and school-based HPV vaccination program increased HPV vaccination rates among medically underserved students in rural middle school districts in Texas by age of initiation	Quasi-experimental study	1766	Adolescents		HPV vaccine initiation and completion		The majority of students initiated the HPV vaccine at 11 (39.5%) and 12 (30.5%). 72.4% of students who received HPV vaccines through the program received them bundled with other vaccines. Those who initiated the HPV vaccine before age 11 had higher UTD percentages	
Rodriguez AM, Do TQN, Eyada MF, Chen L, Schmeler KM, Montealegre JR. Human Papillomavirus Vaccination Uptake in the Rio Grande Valley: Results from a Pilot Community-Based Educational and School-Based Vaccination Program and Its Expansion. Vaccines. 2023;11(2):329. doi:10.3390/vaccines11020329 [117]	2023	South	2016–2022	Assess the effectiveness of a physician-run HPV education campaign and middle school-based HPV vaccination program in rural Texas	Cross-sectional	19,951	Adolescents		HPV vaccine initiation and completion		The overall HPV-UTD was 58.8%. A total of 19,951 students received HPV vaccines either directly or indirectly from the program in the 6 years of intervention. 1549 HPV vaccine initiations, 1042 vaccine completions were delivered at schools throughout the program (total of 2145 students vaccinated directly in the school-based program). 18,172 HPV vaccine initiations and 17,075 HPV vaccine completions were completed through collaborating healthcare practices. Male students and students older at initiation were less likely to be HPV-UTD	
Rosen BL, DiClemente R, Shepard AL, Wilson KL, Fehr SK. Factors associated with school nurses’ HPV vaccine attitudes for school-aged youth. Psychol Health Med Jun. 2017;22(5):535–545. doi:10.1080/13548506.2016.1173710 [118]	2016	National	Not listed	Describe school nurses’ knowledge, perception of role as opinion leader, perceived school district support and attitudes regarding the HPV vaccine for school-aged youth, and determine which factors are associated with positive HPV vaccine attitudes in school nurses	Cross-sectional study	413	(Other) School Nurses	Theory of Planned Behavior	School nurse attitudes towards HPV vaccine initiation		Positive attitudes regarding the HPV vaccine were predicted by higher HPV and vaccine knowledge (β = 0.096, *p* < 0.001) and school nurses’ stronger perceptions of role as opinion leaders for the vaccine (β = 0.665, *p* < 0.001).	
Ryan G, Ashida S, Gilbert PaulA, et al. The Use of Medical Claims Data for Identifying Missed Opportunities for HPV Immunization Among Privately Insured Adolescents in the State of Iowa. J Community Health. 2022;47(5):783–789. doi:10.1007/s10900-022-01110-7 [119]	2022	Midwest	2012–2017	Quantify the number of MOs for HPV vaccination that adolescents experienced between the ages of 11 and 13 using medical claims data and conduct subgroup comparisons by gender and rurality	Cohort study	14,505	Adolescents		Missed opportunities for HPV vaccination	Average number of missed opportunities by subgroup: gender and rurality	16.8% of females and 11.4% of males had completed the vaccine series (3 doses by age 13). Adolescents experienced 5–6 MOs between ages 11 and 13. There were more MOs experienced by non-initiators of the vaccine compared to initiators (7 MOs vs. fewer than 2 MOs).	Urban adolescents experienced more MOs than rural counterparts. Female patients had fewer MOs than males.
Ryan G, Daly E, Askelson N, Pieper F, Seegmiller L, Allred T. Exploring Opportunities to Leverage Pharmacists in Rural Areas to Promote Administration of Human Papillomavirus Vaccine. Prev Chronic Dis. 2020;17. doi:10.5888/pcd17.190351 [120]	2020	Midwest	2018	Assess rural pharmacists’ role in administering and promoting the HPV vaccine in counties in Iowa with low rates of HPV vaccine uptake	Qualitative	11	Other (Pharmacists)		HPV vaccination barriers among rural pharmacists		Pharmacists were willing to administer HPV vaccinations and saw it within their role to do so. Barriers to offering the vaccine included sensitivity of the subject, lack of information, concerns about safety and misinformation	
Ryan GW, Perry SS, Scherer A, et al. Factors contributing to missed opportunities for human papillomavirus vaccination among adolescents, ages 11 to 13, in Iowa. Vaccine X. 2022;11:100192. doi:10.1016/j.jvacx.2022.100192 [121]	2022	Midwest	2012–2020	Explore associations between adolescent and provider characteristics and the number of MOs adolescents experience between ages 11 and 13	Retrospective Cohort study	14,104	Adolescents		Missed opportunities for HPV vaccination		Non-initiators of HPV vaccination had more MOs, fewer well-child visits and fewer other adolescent vaccinations compared to HPV vaccination initiators. MOs decreased with age. Among those who had initiated the vaccine, MOs were higher among those whose PCP was not a pediatrician and those who saw rural providers. Most MOs occurred at acute care visits rather than well-child visits.	
Schrote K, Hersh A, Bruegl A, Rodriguez MI. Women’s perspectives on receiving and expanding access to essential health services in pharmacies in the United States. J Am Pharm Assoc. 2022;62(3):711–716.e3. doi:10.1016/j.japh.2021.11.034 [122]	2022	National	2020	Determine whether there were differences by rurality in women’s perspectives/willingness to receive essential preventative/diagnostic reproductive health services in community pharmacies	Cross-sectional	544	Adults		Women’s perspectives; willingness to receive preventative services in community pharmacies		Women in rural settings were less likely to have reported receiving the HPV vaccine (56.2% vs. 41.7%), and 13.9% of rural respondents reported that they were unsure if they had received the HPV vaccine (*p*= 0.02). Both rural and urban women want to receive preventative reproductive health services in community pharmacies.	
Shah SFA, Ginossar T, Bentley JM, Zimet G, McGrail JP. Using the Theory of Planned behavior to identify correlates of HPV vaccination uptake among college students attending a rural university in Alabama. Vaccine. 2021;39(51):7421–7428. doi:10.1016/j.vaccine.2021.10.082 [123]	2021	South	2019	Examine college students’ intentions to receive the HPV vaccine and examine the relationship between religious beliefs and HPV vaccination uptake status among college students	Cross-sectional survey	257	Adults	Theory of Planned Behavior	Intention to vaccinate		Attitudes and subjective norms were significant predictors of intention to receive vaccinated. Three knowledge statements about HPV and its vaccine were associated with higher attitude scores. The odds of receiving at least one HPV shot were higher for females than for males, for non-Caucasians than for Caucasians. Students who were not vaccinated were more likely to report that religion influenced their health beliefs.	
Shato T, Humble S, Anandarajah A, et al. Influences of sociodemographic characteristics and parental HPV vaccination hesitancy on HPV vaccination coverage in five US states. Vaccine. 2023;41(25):3772–3781. doi:10.1016/j.vaccine.2023.04.082 [124]	2023	Midwest, South	2021	Examine the association of sociodemographic characteristics and HPV vaccination hesitancy with HPV vaccination coverage in five US states with disproportionately low adolescent coverage rates compared to the national average	Cross-sectional survey	926	Adults		HPV vaccination initiation		Children of vaccine hesitant parents were less likely to have received any doses of the HPV vaccine than children of non-vaccine hesitant parents (AOR: 0.17, 95% CI:0.11–0.27). Male children were less likely to have initiated the HPV vaccine series than female children (AOR: 0.70, 95% CI:0.50– 0.97). Older children (13–17 vs. 9–12 years), receiving the meningococcal conjugate or most recent seasonal influenza vaccine were all associated with higher likelihoods of receiving any doses of the HPV vaccine (aOR = 6.01, 95% CI:3.98–9.08; aOR = 2.24, 95% CI:1.27–3.95; aOR = 2.41, 95% CI:1.73–3.36, respectively)	
Song S, White A, Kucik JE. Use of Selected Recommended Clinical Preventive Services—Behavioral Risk Factor Surveillance System, United States, 2018. MMWR Morb Mortal Wkly Rep Apr. 2021;70(13):461–466. doi:10.15585/mmwr.mm7013a1 [125]	2021	National	2018	Ascertain prevalence of the use of selected recommended clinical preventive services among persons aged ≥18 years	Cross-sectional survey	437,436	Adults		HPV vaccine initiation and other preventative services		The overall prevalence of HPV vaccination was 16.5%. There was no statistical difference between rural and urban prevalence for the HPV vaccination (PRR = 1.29, 95% CI: 0.77–2.16, reference = rural). Income was not significant with HPV vaccination prevalence, but having insurance was associated with higher HPV vaccination (PRR = 1.95, 95% CI: 1.17–3.25) compared to uninsured.	
Stewart T, Lee YA, Damiano EA. Do Transgender and Gender Diverse Individuals Receive Adequate Gynecologic Care? An Analysis of a Rural Academic Center. Transgender Health. 2020;5(1):50–58. doi:10.1089/trgh.2019.0037 [126]	2020	Northeast	2015–2018	Compare utilization rates of gynecologic screening services between transgender individuals in a rural setting and cisgender individuals nationally, and determine if utilization rates differed by insurance type or gender identity	retrospective chart review	255	Adults		HPV vaccine initiation		84% (N = 218) of the sample were eligible to receive the HPV vaccination, with 47% (N = 102) receiving the vaccination using the 2018 HPV guidelines. There was a statistically significant difference, with 20% of transgender men, 60% of transgender women, and 60% of GNB/GNC/Genderqueer/gender diverse individuals receiving the vaccination (*p* < 0.001).	
Swiecki-Sikora AL, Henry KA, Kepka D. HPV Vaccination Coverage Among US Teens Across the Rural–Urban Continuum. J Rural Health Sep. 2019;35(4):506–517. doi:10.1111/jrh.12353 [127]	2019	National	2012–2013	Examine associations between HPV vaccination uptake and rural and urban residence, and examine whether vaccine uptake in rural and urban places was modified by area-based poverty	Secondary data analysis	37,115	Adolescents		HPV vaccine initiation and completion		Lower HPV vaccination initiation and completion among teens from isolated small rural towns and small rural towns than among urban teens. Girls from small rural towns had lower odds of completion (OR = 0.74, 95% CI: 0.60–0.91) than girls from urban areas. Boys from isolated small rural towns had statistically significant lower odds of initiation (OR = 0.68, 95% CI: 0.52–0.88) and completion (OR = 0.63, 95% CI: 0.41–0.97) than boys from urban areas.	
Teferra AA, Keller-Hamilton B, Roberts ME, Reiter PL. HPV Vaccine Coverage Among Adolescent Males in Ohio: Results of a Longitudinal Study. Ohio J Public Health. 2019;2(2):15–23. doi:10.18061/ojph.v2i2.9030 [128]	2019	Midwest	2015–2018	Examine HPV vaccine coverage among adolescent males in Ohio and identify predictors of vaccination	Secondary Data Analysis	1126	Adolescents		HPV vaccine initiation	HPV vaccination initiation predictors	42.4% had initiated the HPV vaccine series at the time of the baseline survey. Among sons who were unvaccinated at baseline and whose parents completed a follow-up survey, 36.3% had initiated the HPV vaccine series at follow-up	Initiation was lower among sons of parents with an associate’s degree, or some college education, compared to parents with a high school degree or less (RR = 0.28, 95% CI = 0.46–0.99). Sons whose parents indicated they had received influenza vaccine were more likely to initiate the HPV vaccine series (RR = 1.54, 95% CI = 1.08–2.18), and whose parents indicated a lack of a recent visit to the doctor as a reason for not vaccinating at baseline (RR = 1.41, 95% CI = 1.02–1.95).
Thaker J, Albers AN, Newcomer SR. Nurses’ perceptions, experiences, and practices regarding human papillomavirus vaccination: results from a cross-sectional survey in Montana. BMC Nurs. 2023;22(1). doi:10.1186/s12912-023-01379-6 [129]	2023	Midwest	2020–2021	Determine nurses’ perceptions, experiences, and practices regarding human papillomavirus vaccination in a Rural and medically underserved region of the United States.	Cross-sectional survey	227	Providers		Nurses’ perceptions of clinic vaccination practices & barriers to vaccine uptake & potential strategies to improve HPV vaccination rates	Secondary: Nurses’ report of the estimated percentage of parents who defer HPV vaccination, by age group and sex of adolescent. Tertiary: Nurses’ support of strategies to improve HPV vaccination rates	91.8% (n = 179) of nurses agreed or strongly agreed that it was important that older children and adolescents be vaccinated against HPV, and 89.8% (n = 177) expressed confidence in the safety of the HPV vaccine. Only 34.5% (n = 68) of respondents reported anticipating an uncomfortable conversation while discussing the HPV vaccine with parents of 9 to 12-year-old children.	The highest perceived barriers to recommending and administering the HPV vaccine are parents not thinking that the vaccine is necessary for their sons (n = 146, 74.5%), misinformation that parents receive from the internet or social media (n = 139,71.6%), parental concerns about the safety of the HPV vaccine (n = 132, 67.7%), and irregular well-child visits (n = 130, 66.7%).
Thomaier L, Aase DA, Vogel RI, Parsons HM, Sadak KT, Teoh D. HPV vaccination coverage for pediatric, adolescent and young adult patients receiving care in a childhood cancer survivor program. Prev Med Rep. 2022;29:101972. doi:10.1016/j.pmedr.2022.101972 [130]	2022	Midwest	2014–2019	Determine HPV vaccination coverage among individuals participating in a childhood cancer survivor program (CCSP)	Retrospective cohort study	592	Adolescents and Adults		HPV vaccine initiation		Vaccination initiation among CCSP patients was not statistically significantly different from controls [60.0% vs. 66.3%, OR = 0.82, 95% CI: (0.55, 1.23), *p* = 0.35], and neither was completion (28.5% vs. 30.1%, *p* = 0.09).	
Thomas TL, Caldera M, Maurer J. A short report: parents HPV vaccine knowledge in rural South Florida. Hum Vaccines Immunother. 2019;15(7–8):1666–1671. doi:10.1080/21645515.2019.1600986 [131]	2019	South	2016	Explore parental knowledge and hesitancy of HPV vaccination	Pilot study	123	Parents		HPV vaccine initiation and parental knowledge regarding HPV and the HPV vaccines		Less than 45% of parents/caregivers had vaccinated their child with the HPV vaccine, and 80% of the participants had low or no knowledge of HPV vaccination. Participants with a high school education or less (64%) and conservative religious affiliation, e.g., Baptist and Catholic (74%), did not decline HPV vaccination.	
Thomas TL, Strickland O, Diclemente R, Higgins M. An Opportunity for Cancer Prevention During Preadolescence and Adolescence: Stopping Human Papillomavirus (HPV)-Related Cancer Through HPV Vaccination. J Adolesc Health. 2013;52(5):S60-S68. doi:10.1016/j.jadohealth.2012.08.011 [132]	2013	South	2009–2011	Determine correlates of refusal and acceptance of HPV vaccination by rural parents of preadolescent and adolescent children	Cross-sectional study	519	Parents	Health Belief Model	Intention to vaccinate and HPV vaccine initiation		Being African American and being Baptist lowers the likelihood of parents who choose to vaccinate or intend to vaccinate their children. Parents who had vaccinated or intended to vaccinate had significantly higher scores on perceived barriers (1.02 times more likely to vaccinate) and lower scores on perceived benefits (1.01 times more likely to vaccinate) (model *p* < 0.001).	
Thomas TL, Strickland OL, DiClemente R, Higgins M, Haber M. Rural African American Parents’ Knowledge and Decisions About Human Papillomavirus Vaccination. J Nurs Scholarsh. 2012;44(4):358–367. doi:10.1111/j.1547-5069.2012.01479.x [133]	2014	South	2010–2011	Identify predictors of HPV vaccination among rural African American families, and find culturally specific points of intervention that would increase HPV vaccination rates among children in these communities	Cross-sectional study	400	Parents	Health Belief Model	Intention to vaccinate and HPV vaccine initiation		Intention to vaccinate was significantly different across the three counties (*p* < 0.01). Non-Baptists were 3.6 (95% CI: 2.0–6.6, *p* < 0.001) times more likely to vaccinate compared to Baptists after adjusting for perceived vulnerability and perceived barriers.	
Vamos CA, Kline N, Vázquez-Otero C. Stakeholders’ perspectives on system-level barriers to and facilitators of HPV vaccination among Hispanic migrant farmworkers. Ethn Health Aug. 2022;27(6):1442–1464. doi:10.1080/13557858.2021.1887820 [134]	2021	South	2020	Inform intervention development targeting vaccination uptake and completion, ultimately decreasing HPV-related cancer disparities	Qualitative	13	Other (Stakeholders)	Social Ecological Model, Precede-Proceed Model, CBPR, Intervention Mapping	Stakeholder perceptions of barriers and facilitators to HPV vaccination among Latinx migrant farmworkers		Barriers included lack of healthcare access, language barriers, limited knowledge about HPV and the vaccine, financial constraints, and concerns about immigration status. Facilitators included the presence of outreach programs, culturally tailored interventions, supportive healthcare providers, and social networks within the community that promote vaccination awareness and acceptance.	
Vanderpool RC, Cohen E, Crosby RA, Jones MG, Bates W, Casey BR, Collins T. “1-2-3 Pap” Intervention Improves HPV Vaccine Series Completion among Appalachian Women. J Commun. 2013 Feb;63(1):95–115. doi: 10.1111/jcom.12001. Epub 2013 Jan 10. PMID: 26560123; PMCID: PMC4639462. [135]	2015	South	2010–2011	This study identified correlates of intent to complete the vaccine series and actual series completion. The study tested the efficacy of a DVD intervention to promote series completion.	Randomized controlled trial	344	Adults	Theory of Planned Behavior	HPV vaccine completion		Women’s beliefs that all three doses reduced cancer risk predicted intent and completion. Intention predicted completion, as did the belief that having a friend accompany the woman would promote completion. Beyond these effects, women assigned to the intervention were 2.44 times more likely than women in the control group to complete the series.	
Vielot NA, Lane RM, Loefstedt K, et al. Acceptability and readiness to promote human papillomavirus vaccination at ages 9–10 years: a feasibility study among North Carolina clinics. Pilot Feasibility Stud. 2023;9(1). doi:10.1186/s40814-023-01379-y [136]	2022	South	2022	Assess the feasibility of the age-9 recommendation of HPV vaccination in rural clinics	Pilot study	10	Providers		Attitudes towards recommending HPV vaccination to 9-and 10-year-olds		There are four predominant themes from the interviews: (1) clinics have created opportunities to recommend HPV vaccination during well-child visits; (2) providers educate caregivers who are hesitant about HPV vaccination; (3) providers often consider the benefits of HPV vaccination in the context of adolescent social and physical development; and (4) providers are generally willing and able to promote age-9 HPV vaccination in the clinic.	
Vielot NA, Butler AM, Brookhart MA, Becker-Dreps S, Smith JS. Patterns of Use of Human Papillomavirus and Other Adolescent Vaccines in the United States. J Adolesc Health. 2017;61(3):281–287. doi:10.1016/j.jadohealth.2017.05.016 [137]	2017	National	2009–2014	Describe the patterns of use of universally recommended adolescent vaccines in the United States	Observational Study	1,691,223	Adolescents		HPV vaccine initiation		Only 18.4% of residents received the HPV vaccine compared to Tdap (52.1%) and MenACWY (45.8%). Rural adolescents were less likely than urban adolescents to receive each vaccination except in the Northeast, where they were more likely to receive HPV vaccination (IRR: 1.09, 95% Cl: 1.20–1.13). Timely HPV vaccination was associated with female sex, urbanicity, Western residence, and later birth cohort.	
Walker TY, Elam-Evans LD, Williams CL, et al. Trends in human papillomavirus (HPV) vaccination initiation among adolescents aged 13–17 by metropolitan statistical area (MSA) status, National Immunization Survey—Teen, 2013–2017. Hum Vaccines Immunother. 2019;16(3):554–561. doi:10.1080/21645515.2019.1671765 [138]	2020	National	2013–2017	Examine trends in HPV vaccination initiation coverage by MSA, and examine trends in disparities in HPV vaccination initiation coverage by MSA status over time	Secondary analysis	103,047	Adolescents		HPV vaccine initiation		The five-year average annual percentage point increases in HPV vaccination initiation coverage were similar between MSA designations (4.9–5.2). Coverage was significantly lower among teens living in mostly rural areas, regardless of poverty status, sex, and race/ethnicity, except among black, non-Hispanic adolescents. There was no significant change in the magnitude of the disparity between mostly urban areas and mostly rural areas over time (*p* = 0.98).	
Walker TY, Elam-Evans LD, Yankey D, et al. National, Regional, State, and Selected Local Area Vaccination Coverage Among Adolescents Aged 13–17 Years—United States, 2018. MMWR Morb Mortal Wkly Rep. 2019;68(33):718–723. doi:10.15585/mmwr.mm6833a2 [139]	2019	National	2017- 2018	Examine trends in HPV vaccination initiation coverage by MSA status, and examine trends in disparities in HPV vaccination initiation coverage by MSA status during 2013–2017	Cross-sectional study	103,074	Adolescents		HPV vaccine initiation and completion		In 2018, 51.1% of adolescents aged 13–17 years were up-to-date with the HPV vaccine series, and 68.1% had received ≥1 dose of HPV vaccine. During 2017–2018, the increase in HPV vaccination coverage was attributable to increases among males only.	Small
Warner EL, Fowler B, Martel L, Kepka D. Improving HPV Vaccination Through a Diverse Multi-state Coalition. J Community Health. 2017;42(5):911–920. doi: 10.1007/s10900-017-0334-7 [140]	2017	West	2015–2016	Assess coalition members’ perceptions of barriers and facilitators to HPV vaccination in their communities and evaluate the efficacy, strengths, and future directions of the IWHVC	Mixed methods	122	Adults		HPV vaccination facilitators and barriers		Perceived barriers to vaccination were a lack of education/low knowledge about the HPV vaccine (55.8%), concerns about sexuality/promiscuity (44.2%), and not knowing the vaccine is recommended for boys (38.4%). Top facilitators to HPV vaccination included a strong provider recommendation (53.5%), improved messaging/education (51.2%), and increasing parental buy-in (32.6%).	
Warren BR, Gillette-Walch H, Adler J, et al. Assessment of human papillomavirus vaccination rates of adolescents in California, 2018–2019. Prev Med Rep. 2023;32:102144. doi:10.1016/j.pmedr.2023.102144 [141]	2023	West	2018–2019	Evaluate the vaccine registries (National Immunization Survey (NIS)-Teen, commercial HMOs in California, Medi-Cal, and California Immunization Registry) data for HPV vaccine series completion, and compare their completeness	Secondary data analysis	664,795	Adolescents		HPV vaccination series completion	Secondary: HPV vaccine series completion differences among adolescent females and males. Tertiary: HPV vaccine series initiation versus completion among 13-year-olds by county	HPV series completion among 13-year-olds in 2018 for commercial HMOs was 50%, Medi-Cal was 45%, and the California Immunization Registry was 28%, with NIS-Teen rates for 13 to 17-year-olds at 50% in 2018 and 54% in 2019	Series completion increased for females from 50.1% in 2018 to 61.5% in 2019, but dropped for males from 55.1% to 51.4% in the same time period
Wheeler DC, Miller CA, Do EK, et al. Identifying Area-Level Disparities in Human Papillomavirus Vaccination Coverage Using Geospatial Analysis. Cancer Epidemiol Biomarkers Prev. 2021;30(9):1689–1696. doi:10.1158/1055-9965.epi-21-0331 [142]	2021	South	2010–2018	Determine whether neighborhood sociodemographic variables explain variation in HPV vaccination, and identify areas with significantly depressed vaccination coverage	Secondary data analysis	294,948	Adolescents		HPV vaccine completion		42,145 (28.9%) of girls and 34,760 (23.8%) of boys had completed HPV vaccination; girls had overall higher completion probabilities. Predominantly rural areas had significantly lower vaccination completion rates compared to others.	
Wick JA, Elswick BM. Impact of Pharmacist-Delivered Education on Early Parent Awareness and Perceptions Regarding Human Papillomavirus (HPV) Vaccination in the Community Pharmacy Setting in West Virginia. Innov Pharm. 2018;9(3):8. doi:10.24926/iip.v9i3.1396 [143]	2018	South	2018	Determine parental perceptions of the Human Papillomavirus Vaccine and awareness of vaccine administration at community pharmacies; and describe parental intentions to have children vaccinated against HPV, and assess the impact of pharmacist-led education on these perceptions and intentions	Prospective pretest, post-test study (quasi-experimental)	34	Parents		Intention to vaccinate and awareness of HPV vaccine availability at community pharmacies		Prior to the educational session, 35% of participants planned to vaccinate their child. Following completion of the intervention, 44% of the population intended to vaccinate at the ACIP-recommended age. Participants demonstrated increased awareness of HPV vaccine availability at community pharmacies from 32% (n = 11) to 100% (n = 34).	
Williams CL, Walker TY, Elam-Evans LD. Factors associated with not receiving HPV vaccine among adolescents by metropolitan statistical area status, United States, National Immunization Survey-Teen, 2016–2017. Hum Vaccin Immunother Mar. 2020;16(3):562–572. doi:10.1080/21645515.2019.1670036 [144]	2020	National	2016–2017	Identify sociodemographic factors associated with not initiating the HPV vaccine series, and determine whether these factors differed by MSA status	Secondary data analysis	41,424	Adolescents		Non-initiation of the HPV vaccine series		A significantly higher percentage of suburban (39.2%) and mostly rural (45.4%) teens had not received any doses of the HPV vaccine compared to mostly urban teens (32.0%). Regardless of MSA designation, factors for not receiving HPV included living in the South, having a mother with some college education, not having an 11–12-year-old well-child visit, and not receiving a provider recommendation for vaccination. There was no difference in the percentage of mostly rural teens (78.9%) with missed opportunities for HPV vaccination when compared to mostly urban teens (79.3%).	
Yoost JL, Starcher RW, King-Mallory RA, Hussain N, Hensley CA, Gress TW. The Use of Telehealth to Teach Reproductive Health to Female Rural High School Students. J Pediatr Adolesc Gynecol. 2017;30(2):193–198. doi:10.1016/j.jpag.2016.10.002 [145]	2017	South	2015	Evaluate the use of telehealth to teach reproductive health in rural areas with high rates of teen pregnancy	Prospective cohort study	55	Adolescents		HPV vaccine initiation		Those reporting vaccine initiation or completion was 38% (10/26) at the time of the educational session post-test. This report increased to 71.4%, 15/21 (*p* = 0.03) at 6 months among those who attended that session and increased to 70%, 26/37 (*p* = 0.001) among all subjects who completed the 6-month survey (n = 37).	
Zahnd WE, Harrison SE, Stephens HC, et al. Expanding access to HPV vaccination in South Carolina through community pharmacies: A geospatial analysis. J Am Pharm Assoc. 2020;60(6):e153-e157. doi:10.1016/j.japh.2020.05.005 [146]	2020	South	2019	Determine whether spatial access to pharmacies among adolescents and young adults in South Carolina varied by rurality and geographic access to primary care providers	Secondary data analysis	1010	Pharmacies (other)		Spatial access to pharmacies		There were statistically significantly higher spatial accessibility scores in non-HPSA–designated CTs across South Carolina as a whole, as well as in both metropolitan and rural and small-town areas. However, among CTs in micropolitan areas, no difference in spatial accessibility scores was found between HPSA-designated and non-HPSA–designated CTs.	
Zhang J, Xue H, Calabrese C, Chen H, Dang JHT. Understanding Human Papillomavirus Vaccine Promotions and Hesitancy in Northern California Through Examining Public Facebook Pages and Groups. Front Digit Health. 2021;3. doi:10.3389/fdgth.2021.683090 [147]	2021	West	2010–2021	Understand HPV vaccine promotions and hesitancy in Northern California by examining public Facebook pages and groups	Observational study	212	Adults		Sentiments, Negative emotions, and Thematic Topics in Facebook posts and comments		There was significantly more positive sentiment in comments than in posts, more negative sentiment in comments than in posts, and more anger in comments than in posts. Post themes included awareness and screening of HPV and cervical cancer, STI testing services, information sources, and calls to action for health services. Comment themes were related to vaccine hesitancy, discussing vaccine risks, safety concerns, and distrust in vaccine science, citing misinformation. When comparing high-coverage counties, there were no significant differences across all dimensions of sentiment and emotions for posts. For comments, there was a significantly higher level of anger in high-coverage counties than in low-coverage counties.	
Zoellner JM, Porter KJ, Brock DJP, et al. Advancing engagement and capacity for rural cancer control: a mixed methods case study of a Community-Academic Advisory Board in the Appalachia region of Southwest Virginia. Res Involv Engagem. 2021;7(1). doi:10.1186/s40900-021-00285-y [148]	2021	South	2017–2020	Describe engagement processes used to prioritize and address regional comprehensive cancer control needs among a Community-Academic Advisory Board (CAB) in the medically underserved, rural Appalachian region	Convergent parallel mixed methods	69	Other	Community-Based Participatory Research, Comprehensive Participatory Planning and Evaluation	Habits of community advisory boards, challenges, and strengths across those habits		Across habits and at both Time 2 and Time 3 interviews, strengths reported by CAB members outweighed the challenges in both quantity and frequency. An exception was for diversified funding, where reported strengths and challenges were relatively more even. Related to challenges, limited time was consistently mentioned across most habits and was viewed as a limiting factor at both time points. Also, implications of COVID-19, especially as it related to effective communication and diversified funding, emerged as a major challenge at Time 3.	
* These papers (as well as Chido-Amajuoyi et al., 2022, reference [37]) rely on one statewide cross-sectional survey of healthcare professionals in Texas (N = 1283).												

### Description of Process Measures in Implementation and Intervention Studies (N = 20)

**Reference**	**General Description of Intervention**	**Study Type**	**Intervention Type**	**Free Vaccination Offered**	**Intervention Level**	**Intended Audience for Intervention**	**Intervention Duration**	**Duration of Training, Educational Sessions**	**Number of Sessions; Number of media Outlets, Flyers**	**Mode of Intervention**	**Who Intervened (Directly Performed Intervention)**
Crosby RA, Casey BR, Vanderpool R, Collins T, Moore GR. Uptake of free HPV vaccination among young women: a comparison of rural versus urban rates. J Rural Health Winter. 2011;27(4):380–384. doi:10.1111/j.1748-0361.2010.00354.x [27]	Young rural women attending rural clinics (n = 246), young women attending a rural community college (n = 251) and young women attending an urban university health clinic (n = 209) were recruited in Kentucky. After completing a brief questionnaire, women received a free voucher for HPV vaccination.	Interventional	Vaccine voucher	Yes	Individual-level	College women	23 months	N/A	N/A	In-person	Research assistant
Beck A, Bianchi A, Showalter D. Evidence-Based Practice Model to Increase Human Papillomavirus Vaccine Uptake: A Stepwise Approach. Nurs Womens Health. 2021;25(6):430–436. doi:10.1016/j.nwh.2021.09.006 [59]	Education targeting parental HPV vaccine hesitancy and strong recommendations for immunization was administered by healthcare providers to parents of youth and adolescents who are vaccine-eligible.	Interventional	Parental Education	No	Clinic	Parents of unvaccinated children from 11 to 17	6 weeks (additional 6 weeks for control period)	N/A	N/A	In-person	Clinic staff and vaccine providers
Berenson AB, Hirth JM, Kuo YF, Rupp RE. Quantitative and qualitative assessment of an all-inclusive postpartum human papillomavirus vaccination program. Am J Obstet Gynecol. 2021;224(5):504.e1–504.e9. doi:10.1016/j.ajog.2020.11.033 [61]	Postpartum women eligible for HPV vaccine were offered education on the HPV vaccination and the CDC facts sheet. Patients who gave consent were administered a dose prior to discharge, or were scheduled for outpatient vaccination due to time purposes with follow-up doses along with the postpartum doctor visits.	Interventional	Patient Education	Yes	Individual-level	Postpartum women	3 years (evaluation portion of intervention)	N/A	N/A	In-person	Patient Navigators
Brewer NT, Hall ME, Malo TL, Gilkey MB, Quinn B, Lathren C. Announcements Versus Conversations to Improve HPV Vaccination Coverage: A Randomized Trial. Pediatrics. 2017;139(1). doi:10.1542/peds.2016-1764 [66]	Randomized clinics to receive no training (control), announcement training, or conversation training.	Interventional	Vaccine provider education	No	Providers	Vaccine providers	4 months	1 h	1 training session	In-person	physician educator
Carman AL, McGladrey ML, Goodman Hoover A, Crosby RA. Organizational Variation in Implementation of an Evidence-Based Human Papillomavirus Intervention. Am J Prev Med. 2015;49(2):301–308. doi:10.1016/j.amepre.2015.03.011 [70]	Pragmatic implementation study focused on LHDs the option of showing the HPV vaccine informational video after the first dose of the vaccine but also pilot tested the feasibility and acceptability of other delivery options	Implementation	Patient Education (Implementation at LHD clinics)	No	Clinic-level * (local public health department clinics)	Local health department clinics	6 months	N/A	N/A	Unspecified (mainly remote and indirect)	Mainly self-directed (Research team gave general guidelines)
Cates JR, Shafer A, Diehl SJ, Deal AM. Evaluating a County-Sponsored Social Marketing Campaign to Increase Mothers’ Initiation of HPV Vaccine for Their Preteen Daughters in a Primarily Rural Area. Soc Mark Q. 2011;17(1):4–26. doi:10.1080/15245004.2010.546943 [73]	They placed posters and brochures in English and/or Spanish with a goal of one location for every ten mothers of 11–12-year-old girls in each city in the four counties, according to census data. On launch date and six weeks later, media releases about the campaign were sent to 10 newspapers and PSAs were sent to 15 radio or television stations.	Interventional	HPV Awareness/Media Campaign	No	Community social marketing campaign	Mothers of 11–12 y/o girls	3 months	N/A	10 newspapers and 15 radio/TV stations were used in the campaign (sent media on launch date and then 6 weeks later)	Indirect	Research team
Dang JHT, McClure S, Gori ACT. Implementation and evaluation of a multilevel intervention to increase uptake of the human papillomavirus vaccine among rural adolescents. J Rural Health Jan. 2023;39(1):136–141. doi:10.1111/jrh.12690 [76]	There was intervention strategies applied on three levels. On the parent level there was tailored HPV vaccination reminder postcards sent out. On the primary care team level there were 3 clinic-wide HPV vaccination trainings and a quarterly review of HPV vaccination data. On the clinic level there was a physician champion (the clinic’s Medical Director) and clinic visual cues (examination room posters, educational handouts, lanyards, and pins).	Interventional	Other (Parental-education/awareness, Clinic-awareness; Provider Education)	No	Multilevel	clinics, primary care providers, and parents/guardians	1.5 years	1 h	3 sessions for PCPs	In-person (PCPs), indirect (parental postcards)	Research team
Daniel CL, Lawson F, Vickers M, et al. Enrolling a rural community pharmacy as a Vaccines for Children provider to increase HPV vaccination: a feasibility study. BMC Public Health. 2021;21(1). doi:10.1186/s12889-021-11304-8 [77]	Enrolled a rural community pharmacy as a Vaccines for Children (VFC) provider to provide free vaccines to eligible adolescents. Development and execution of a health communication campaign for the community	Feasibility-Interventional	Other (Pharmacy VFC enrollment)	VFC-eligible only	Community	Pharmacy and surrounding community	12 months	N/A	N/A	Both	Research team
Ford M, Cartmell K, Malek A, et al. Evaluation of the First-Year Data from an HPV Vaccination Van Program in South Carolina, U.S. J Clin Med. 2023;12(4):1362. doi:10.3390/jcm12041362 [80]	HPV Vaccination Van Program; conducted a town hall meeting prior to the HPV vaccine clinic in their district with several speakers who spoke about their experience (a teacher who had HPV, her son telling why he received it, a physician telling facts about the vaccine). Putting vaccination clinics at school and sharing the video of the town hall meeting.	Interventional	Community education/awareness	VFC-eligible only	Community-level	Adolescents 13–17	1 year	1 h	1 town hall	virtual	town hall speakers: science teacher, her son, 2 physicians from nearby medical school
Harry ML, Asche SE, Freitag LA, et al. Human Papillomavirus vaccination clinical decision support for young adults in an upper midwestern healthcare system: a clinic cluster-randomized control trial. Hum Vaccines Immunother. 2022;18(1). doi:10.1080/21645515.2022.2040933 [85]	Randomized control trial to test clinical decision support among 34 clinics with three treatment arms: Clinical Decision Support only, Clinical Decision Support with Shared Decision-Making Tools, and Usual Care over 12 months from first visit by eligible patients	Interventional	Vaccine provider education	No	Clinic-level	Clinic staff	20 months	unspecified	4 sessions (2 in-peron, 2 virtual webinars)	Both	research team (for staff education)
Jafari SDG, Appel SJ, Shorter DG. Risk Reduction Interventions for Human Papillomavirus in Rural Maryland. J Dr Nurs Pract. 2020;13(2):134–141. doi:10.1891/jdnp-d-19-00047 [88]	Advertisement of a film screening event was undertaken via flyers on the college campus, in the office, and through social media posts. Women’s Health clinic office staff were instructed to review HPV immunization records at the time of the annual Well Woman Visit for females aged 12–26 years. The CDC HPV education sheet was distributed to parents of those aged 12–17 and to patients aged 18–26 years. For the public awareness campaign, screenings of the documentary Someone You Love; The HPV Epidemic © by Lumiere Media Inc. (2015), were undertaken with permission. The screening served as the focal point for part one of this initiative. The screenings were heavily advertised at the local community college and on social media. The second component of the project measured the impact of patient education.	Interventional	Community education/awareness	No	Individual-level	Parents of eligible children and adult patients	Unspecified	N/A	unspecified	Both	Research team
Kepka D, Christini K, McGough E, et al. Successful Multi-Level HPV Vaccination Intervention at a Rural Healthcare Center in the Era of COVID-19. Front Digit Health. 2021;3. doi:10.3389/fdgth.2021.719138 [89]	Human papillomavirus vaccination training for the healthcare team included two 1 h early morning video calls that focused on training providers and support staff at TMC on evidence-based HPV vaccination systems, vaccine recommendations, and patient education materials relevant to their patient population. Healthcare team members were given evidence-based patient center HPV vaccination education materials. An HPV vaccination reminder campaign was performed for patients/caregivers with age-eligible children for the HPV vaccine (children ages 11–17) and young adults (ages 18–26) who are also age-eligible for the HPV vaccine.	Interventional	Vaccine provider education	No	Multilevel	Vaccine providers	2019–2021	1 h	2 main training sessions (1 optional/refresher)	virtual	Research team
Kepka D, Coronado GD, Rodriguez HP, Thompson B. Evaluation of a Radionovela to Promote HPV Vaccine Awareness and Knowledge Among Hispanic Parents. J Community Health. 2011;36(6):957–965. doi:10.1007/s10900-011-9395-1 [90]	Intervention Arm: The radionovela addresses facts about cervical cancer, HPV, and the HPV vaccine, concerns about the HPV vaccine, and decision-making activities related to vaccine uptake. Control Arm: the same 5 min of Spanish radio programming prior to the control message, but included public service announcement related to prostate cancer prevention	Interventional	HPV Awareness/Media Campaign	No	Individual-level	Parents of female children (9–17 y/o)	3 months	N/A	1 listening activity	In-person	Research team and local health educators
Panagides R, Voges N, Oliver J, Bridwell D, Mitchell E. Determining the Impact of a Community-Based Intervention on Knowledge Gained and Attitudes Towards the HPV Vaccine in Virginia. J Cancer Educ Apr. 2023;38(2):646–651. doi:10.1007/s13187-022-02169-5 [108]	Showed documentary “Someone You Love: The HPV Epidemic” and evaluated intention to vaccinate and HPV knowledge through surveys before and after participants watched the film.	Interventional	Community education/awareness	No	Individual-level	18+ adults in community	2016–2019	unspecified	>1 movie showing	In-person	Research team
Paskett ED, Krok-Schoen JL, Pennell ML, et al. Results of a Multilevel Intervention Trial to Increase Human Papillomavirus (HPV) Vaccine Uptake among Adolescent Girls. Cancer Epidemiol Biomarkers Prev. 2016;25(4):593–602. doi:10.1158/1055-9965.epi-15-1243 [109]	Clinical level: posters, brochures, and tabletop tent cards for the HPV vaccine intervention. Provider level: For the HPV vaccine education session, we modified an evidence-based tobacco cessation program (38) focused on the “5 A’s” and “5 R’s.” The session was on current evidence-based HPV vaccine information and strategies designed to assist physicians in discussing HPV vaccination with parents Parent level: an educational brochure and DVD video about HPV and HPV vaccination, a magnet reminder to receive the 2nd and 3rd HPV vaccine shots, and a Centers for Disease Control and Prevention (CDC) HPV vaccine information statement. (Provider control: Providers were given information on the flu and flu vaccine. Parent level Control: The comparison group was mailed a packet that included similar items, a flu vaccine information statement from the CDC and flu information sheets from Ohio Department of Health.)	Interventional	Other (Clinic/Provider/Parental education)	No	Multilevel	Vaccine providers and parents	12 months (provider), 6 months (measuring secondary outcomes in patients)	1 h (provider education)	1 educational session for providers	indirect (parent/clinic)	Research team
Rodriguez AM, Do TQN, Chen L, Schmeler KM, Montealegre JR, Kuo YF. Human papillomavirus vaccinations at recommended ages: How a middle school-based educational and vaccination program increased uptake in the Rio Grande Valley. Hum Vaccines Immunother. 2022;18(6). doi:10.1080/21645515.2022.2133315 [116]	A comprehensive school-based intervention was conducted to encourage middle school students to become vaccinated for HPV. Several school districts participated in the intervention that included physician-led educational events about HPV and its vaccine, five school-based vaccination events at participating schools (prior to COVID-19), and remote/outdoor events (during COVID-19).	Interventional	Community education/awareness	Yes	Individual-level/Community-level	middle schoolers	2016–2022	unspecified	5 school vaccination events; unspecified number of physician-led educational events	In-person (some adaptations during the pandemic)	Physician-led educational events
Rodriguez AM, Do TQN, Eyada MF, Chen L, Schmeler KM, Montealegre JR. Human Papillomavirus Vaccination Uptake in the Rio Grande Valley: Results from a Pilot Community-Based Educational and School-Based Vaccination Program and Its Expansion. Vaccines. 2023;11(2):329. doi:10.3390/vaccines11020329 [117]	A comprehensive school-based intervention was conducted to encourage middle school students to become vaccinated for HPV. Several school districts participated in the intervention that included physician-led educational events about HPV and its vaccine, five school-based vaccination events at participating schools (prior to COVID-19), and remote/outdoor events (during COVID-19).	Interventional	Community education/awareness	Yes	Individual-level/Community-level	middle schoolers	2016–2022	unspecified	5 school vaccination events; unspecified number of physician-led educational events	In-person (some adaptations during the pandemic)	Physician-led educational events
Vanderpool RC, Cohen E, Crosby RA, Jones MG, Bates W, Casey BR, Collins T. “1-2-3 Pap” Intervention Improves HPV Vaccine Series Completion among Appalachian Women. J Commun. 2013 Feb;63(1):95–115. doi: 10.1111/jcom.12001. Epub 2013 Jan 10. PMID: 26560123; PMCID: PMC4639462. [135]	Women in the intervention group viewed a 13 min educational DVD, called “1–2–3 Pap.” The DVD focused on the importance of HPV vaccination and guideline-concordant Pap testing for Appalachian Kentucky women.	Interventional	HPV Awareness/Media Campaign	Yes * (only first dose was free, the rest of series was the participants’ financial obligation)	Individual-level	Women (18–26)	1 year	13 min video (for intervention arm)	1 video or pamphlet session (13 min for intervention video)	Unspecified	Self-directed
Wick JA, Elswick BM. Impact of Pharmacist-Delivered Education on Early Parent Awareness and Perceptions Regarding Human Papillomavirus (HPV) Vaccination in the Community Pharmacy Setting in West Virginia. Innov Pharm. 2018;9(3):8. doi:10.24926/iip.v9i3.1396 [143]	An educational session regarding HPV vaccination	Interventional	Parental Education	No	Individual-level	Parents of children under 9	5 months	30 min	1 session per parent (4 sessions held)	In-person	Pharmacist
Yoost JL, Starcher RW, King-Mallory RA, Hussain N, Hensley CA, Gress TW. The Use of Telehealth to Teach Reproductive Health to Female Rural High School Students. J Pediatr Adolesc Gynecol. 2017;30(2):193–198. doi:10.1016/j.jpag.2016.10.002 [145]	Teleconferencing equipment connected rural high schools to a distant academic institution. Telehealth sessions included reproductive health and life skills topics. Demographic information, session pre- and post-tests, and 6-month assessment were obtained.	Interventional	Patient Education	No	Individual-level	Female high school students	4 weeks	1 h	8 sessions	Virtual	Faculty/residents/medical students 3 h from study locations
* These papers (as well as Chido-Amajuoyi et al., 2022, reference [37]) rely on one statewide cross-sectional survey of healthcare professionals in Texas (N = 1283).											
